# Structure, mechanism, and regulation of mitochondrial DNA transcription initiation

**DOI:** 10.1074/jbc.REV120.011202

**Published:** 2021-01-13

**Authors:** Urmimala Basu, Alicia M. Bostwick, Kalyan Das, Kristin E. Dittenhafer-Reed, Smita S. Patel

**Affiliations:** 1 Department of Biochemistry and Molecular Biology, Rutgers Robert Wood Johnson Medical School, Piscataway, New Jersey, USA; 2 Graduate School of Biomedical Sciences, Rutgers Robert Wood Johnson Medical School, Piscataway, New Jersey, USA; 3 Department of Chemistry, Hope College, Holland, Michigan, USA; 4 Department of Microbiology, Immunology, and Transplantation, Rega Institute for Medical Research, KU Leuven, Leuven, Belgium

**Keywords:** mitochondria, transcription, RNA polymerase, structure-function, transcription initiation factors, transcription regulation, enzyme mechanism, mitochondrial DNA (mtDNA), DNA transcription, enzyme structure, human mitochondrial RNA polymerase, mitochondrial DNA transcription, mitochondrial gene regulation, yeast mitochondrial RNA polymerase

## Abstract

Mitochondria are specialized compartments that produce requisite ATP to fuel cellular functions and serve as centers of metabolite processing, cellular signaling, and apoptosis. To accomplish these roles, mitochondria rely on the genetic information in their small genome (mitochondrial DNA) and the nucleus. A growing appreciation for mitochondria's role in a myriad of human diseases, including inherited genetic disorders, degenerative diseases, inflammation, and cancer, has fueled the study of biochemical mechanisms that control mitochondrial function. The mitochondrial transcriptional machinery is different from nuclear machinery. The *in vitro* re-constituted transcriptional complexes of *Saccharomyces cerevisiae* (yeast) and humans, aided with high-resolution structures and biochemical characterizations, have provided a deeper understanding of the mechanism and regulation of mitochondrial DNA transcription. In this review, we will discuss recent advances in the structure and mechanism of mitochondrial transcription initiation. We will follow up with recent discoveries and formative findings regarding the regulatory events that control mitochondrial DNA transcription, focusing on those involved in cross-talk between the mitochondria and nucleus.

## Brief overview of mitochondrial functions

Mitochondria are at the heart of energy production in eukaryotic cells, producing and regulating ATP production through the oxidative phosphorylation (OXPHOS) pathway. Mitochondria are hypothesized to originate from an endosymbiotic event occurring ∼1.5 billion years ago in which an archaea-type host engulfed an α-proteobacterium–like ancestor. This hypothesis stems from analyses of mitochondrial genes and their genomic organization and distribution (1, 2, 3). The endosymbiotic event equipped the host with “compartmentalized bioenergetic and biosynthetic factories” (1). At the same time, the endosymbiont acquired access to various metabolites from the host. Gene transfer events throughout evolution have led to a division of mitochondrial genetic information between the nucleus and the mitochondria (4).

Intriguingly, mitochondria have retained their small genome throughout evolution. Human mitochondrial DNA (mtDNA) was the first genome to be completely sequenced in 1981 (5). It is a 16.5-kb circular dsDNA lacking introns and residing within the mitochondrial matrix. The *Saccharomyces cerevisiae* (yeast) mtDNA is an 85-kb linear DNA that was sequenced in 1998 (6) and shown to contain introns (6, 7, 8), unlike h-mtDNA that lacks introns. The h-mtDNA codes for 22 tRNAs, two rRNAs, and 13 mRNAs that encode essential OXPHOS protein subunits (5). The yeast mtDNA codes for 24 tRNAs, two rRNAs, and only eight mRNAs to make seven OXPHOS subunits and one ribosomal subunit (6). The remaining OXPHOS protein subunits (77 in humans) and ∼1,500 other mitochondrial proteins, including the proteins that maintain and express the mtDNA, are encoded by genes in the nucleus. Therefore, mitochondria rely on the nucleus to function correctly, and communication and coordination between the transcription events in mitochondria and nucleus are crucial for oxidative ATP production and mitochondrial homeostasis.

Even though mitochondria have a prokaryotic origin, the mitochondrial replication and transcription machinery is similar to that of bacteriophages (9). The core RNA polymerase (RNAP) subunit that catalyzes mtDNA transcription in the mitochondria belongs to the single-subunit class of RNAPs and structurally homologous bacteriophage T7 RNAP, with the exception that the mtRNAPs depend on transcription factors. The more complex organization of mtRNAPs likely evolved to provide additional points of regulation to respond appropriately to the cell's energy needs. Our understanding of the transcription mechanism by mtRNAPs lags behind our knowledge of bacterial and nuclear DNA transcription. Most of our understanding of the mechanism of mtDNA transcription is derived from studies of the yeast *S. cerevisiae* and human mtRNAP complexes, and in several aspects, yeast has remained a model system for biochemistry and genetics (10). Both transcriptional complexes are successfully reconstituted *in vitro*, which has aided their analysis through biochemical and recent high-resolution structural studies. This review will provide recent insights into the mechanism of transcription initiation by mtRNAPs with a parallel discussion of yeast and human systems. The similarities will bring out the underlying conserved mechanisms, and the differences will reveal the additional layers of regulatory measures present in the human system. The detailed view of transcription initiation will be complemented by a comprehensive picture that will highlight multiple feedback events between the mitochondria and nucleus that are necessary for regulating mtDNA transcription. Mammalian mtDNA transcription and regulation has been reviewed in many excellent articles (11, 12, 13, 14). Herein, we will highlight recent studies that further enhance our understanding of the regulation of mammalian mtDNA transcription. The regulatory mechanism of *S. cerevisiae* mtDNA transcription may differ from mammalian systems and have been discussed elsewhere (15, 16, 17).

## The mitochondrial transcription initiation machinery

The mitochondria's transcription machinery is a multicomponent system consisting of the catalytic mtRNAP subunit and several accessory transcription factors. This machinery catalyzes all the major transcription stages, including promoter recognition, promoter-specific transcription initiation, elongation, and termination. Each of these events and the steps within them is subject to regulation. The human mtRNAP (h-mtRNAP) is encoded by *POLRMT,* which needs two transcription factors, TFB2M and TFAM, for promoter-specific transcription initiation. The yeast mtRNAP (y-mtRNAP) is encoded by *RPO41,* and it requires only one initiation factor, MTF1, for promoter-specific transcription. All of the transcriptional machinery proteins are nuclear-encoded and have a 20–30-amino acid mitochondrial localization sequence for entry into the mitochondria. In both yeast and human mitochondrial systems, a successful transcription event produces a polycistronic RNA transcript, processed to produce mature RNAs. In literature, human mtRNAP is referred to by its gene name, POLRMT or h-mtRNAP, whereas the yeast mtRNAP is referred to as RPO41 mostly.

### Mitochondrial DNA promoters

**Figure 1:**
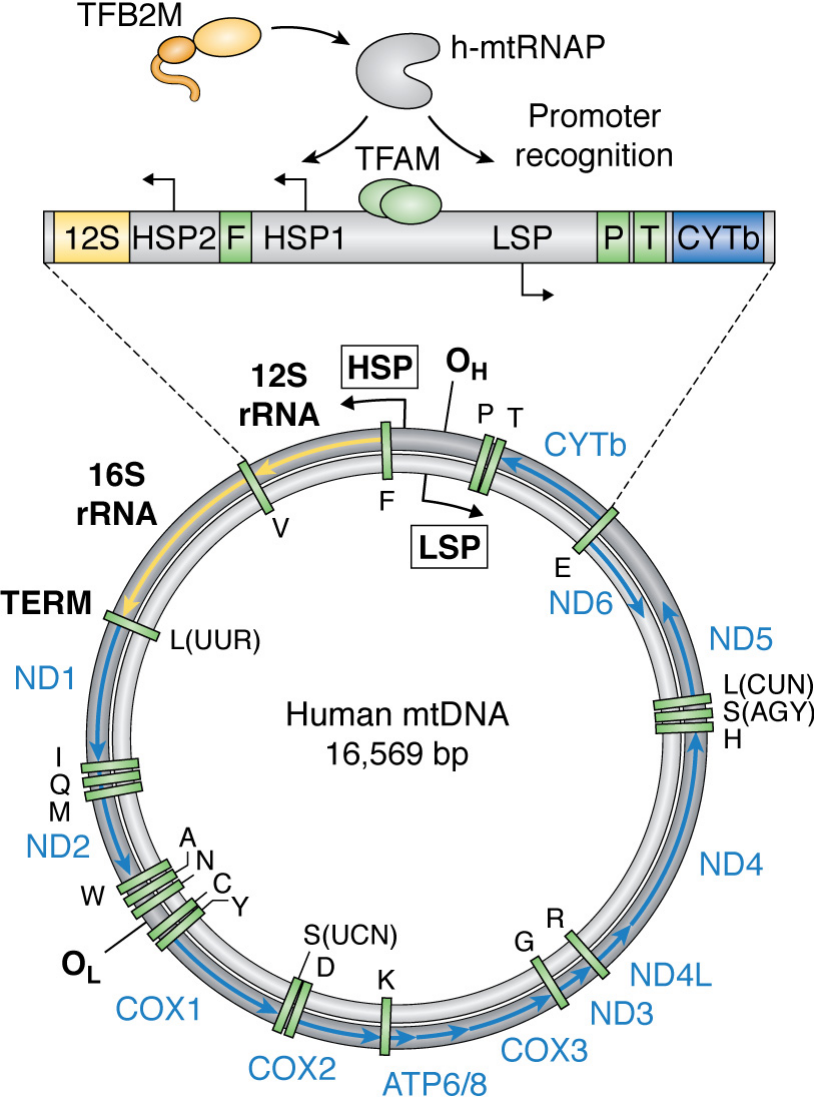
**Human mitochondrial DNA composition.** Human mtDNA is depicted with a heavy strand in *black* and light strand in *gray*. rRNAs (*yellow*), mRNAs (*blue*), and tRNAs (*green*) are *labeled*. Transcription is bidirectional and initiated in the D-loop control region (shown *expanded*) from three promoters, HSP1, HSP2, and LSP. TFAM (*pale green*) binds mtDNA upstream of promoters, recruiting TFB2M (*orange*) and h-mtRNAP (*gray*) to initiate transcription.

**Figure 2:**
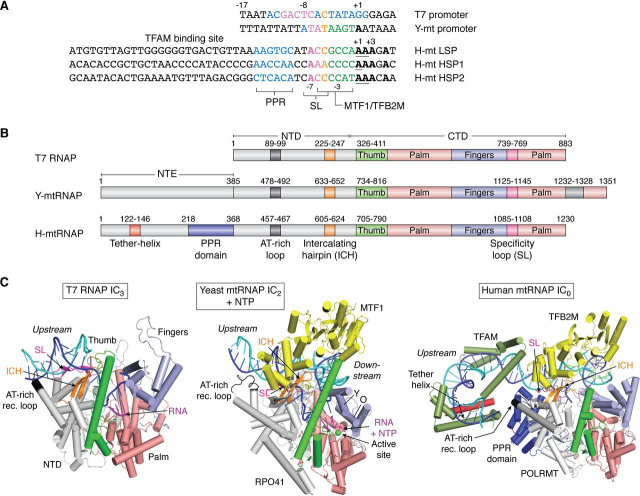
**Comparison of single-subunit RNAP promoters and protein structures.***A*, the DNA promoter sequence (nontemplate strand) of phage T7, yeast, and human mtDNA. The conserved nucleotides within the promoter region are in *boldface type*. T7 promoters are conserved from −17 to +2, y-mtDNA promoters are conserved from −8 to +1, and h-mtDNA promoters have conserved −7, −3, +1 to +3, and +5 base pairs. *B*, the domain structures of T7 RNAP, y-mtRNAP, and h-mtRNAP. The *color-coded regions* show conserved elements in the CTD and the NTD. An N-terminal extension (NTE) is present in mtRNAPs but lacking in T7 RNAP. *C*, high-resolution structures of the initiation complexes of T7 RNAP with 3-bp RNA:DNA (PDB entry 1QLN), yeast mtRNAP with 2-bp RNA:DNA and the next NTP (PDB entry 6YMW), and human mtRNAP without NTP (PDB entry 6ERP) are shown. The conserved elements in the three RNAPs are *color-coded* and *labeled*. The template DNA is shown in *blue*, nontemplate DNA in *cyan*, and RNA in *magenta*. The Y-helix and O-helix in the fingers domain in y-mtRNAP are labeled as *Y* and *O*, respectively.

The 16.5-kb circular mammalian mtDNA molecule (Fig. 1) consists of a light-strand and a heavy-strand DNA, distinguished by GC content (18). Both strands encode various mitochondrial mRNA, rRNA, and tRNA genes. The only noncoding region in the human mtDNA is a 1.1-kb region, a portion (∼650 bp) of which contains a unique three-stranded DNA loop structure (D-loop) (19). The control elements for transcription and replication of mtDNA, including the light-strand promoter (LSP) and the heavy-strand promoters (HSP1 and HSP2), are present within a short ∼250-bp segment of a noncoding region adjacent to the D-loop. The LSP and HSP promoters transcribe h-mtDNA in opposite directions. The 154-bp region between the LSP and HSP1 promoters can bind several TFAM molecules and regulate the two promoters (20). The LSP drives one mRNA to code an OXPHOS protein and eight tRNAs and is responsible for making the primer that initiates leading strand mtDNA replication. The closely spaced HSP1 and HSP2 promoters in the heavy strand drive the expression of 12 mRNAs coding for OXPHOS proteins, two rRNAs, and 14 tRNAs of the mitochondrial ribosome. Early studies identified the transcription start sites on the light and heavy strands of h-mtDNA (21, 22). The transcription start site of LSP is at position 407/408, that of HSP1 is at position 561, and the HSP2 transcription start site is likely at position 643/644 (23, 24) (Fig. 2*A*). Analysis of nascent transcripts by PRO-Seq and GRO-Seq methods confirmed the transcription initiation sites on h-mtDNA and showed quantitative differences between light- and heavy-strand transcription efficiency among different human cell lines (25).

A comparison of LSP, HSP1, and HSP2 promoter sequences reveals a mild consensus in the base pairs around the transcription initiation site (Fig. 2*A*). The −7 A, −3 C, +1 to +3 AAA, and +5 A are conserved positions in all three promoters. This region interacts with TFB2M, which is required for transcription by all three promoters. The significant differences in the transcription initiation mechanism at the three promoters likely arise from the promoter sequences' differences and their requirement for TFAM. *In vitro* studies indicate that LSP and HSP1 require TFAM for optimal transcription (26, 27), whereas TFAM inhibits transcription from HSP2 (23, 24). TFAM at high concentrations also inhibits LSP and HSP1 promoters (20, 28). Interestingly, DNA supercoiling activates TFAM-independent transcription from HSP1 and HSP2, but not from LSP (29).

In contrast to the human system, the yeast *S. cerevisiae* mtDNA is a noncircular AT-rich DNA. There are 11 mitochondrial DNA promoters spread across the genome that account for the expression of all genes and three origins of replication (30). Additionally, recombination-dependent replication initiation is prevalent in yeast (8, 31). The y-mtDNA promoters contain a consensus nonanucleotide sequence (Fig. 2*A*), which interestingly is conserved in promoters of the distantly related yeast *Kluyveromyces lactis* (32, 33).

### Mitochondrial RNA polymerases

The h-mtRNAP and y-mtRNAP show a significant structural and amino acid sequence similarity to each other and T7 RNAP (9) (Fig. 2*B*). The ∼800 amino acids of the C-terminal domain (CTD) of y-mtRNAP and T7 RNAP have a 28% sequence identity, and there is 41% sequence identity between y-mtRNAP and h-mtRNAP. The CTD's basic structure resembles the classic “right hand” shape composed of the thumb, palm, and fingers domains (Fig. 2*C*) (34, 35, 36, 37, 38, 39). The palm domain and fingers domain contain the polymerase active site responsible for catalyzing nucleotide incorporation, and the thumb domain is essential for DNA binding (40). The y-mtRNAP and h-mtRNAP are active in RNA synthesis on single-stranded templates and bubble DNAs (41, 42), but they need transcription factors to catalyze RNA synthesis on duplex promoter DNAs. The N-terminal domain (NTD) in the single-subunit RNAPs contains the promoter-recognizing structural elements like the AT-rich recognition loop and the intercalating hairpin (ICH); the third such element, the specificity loop, is in the CTD. The promoter-binding elements have diverged between T7 RNAP and mtRNAPs, which correlates with loss in promoter sequence conservation and increasing reliance on transcription factors. T7 promoters contain a 23-bp conserved sequence, y-mtDNA promoters have a 9-bp conserved sequence, and there are very few conserved base pairs in h-mtDNA promoters (Fig. 2*A*). The promoter-binding elements in T7 RNAP make extensive base-specific interactions with the T7 promoter, and T7 RNAP does not require transcription factors. The promoter-binding elements in mtRNAPs show fewer base-specific interactions and more reliance on initiation factors for promoter-specific transcription. The dependence of mtRNAPs on transcription factors adds layers of gene regulation that is necessary to synchronize mitochondrial energy production to cellular demand.

The mtRNAPs contain an N-terminal extension (NTE), which is not present in T7 RNAP. The NTE in h-mtRNAP harbors two pentatricopeptide repeat (PPR) domains that interact with the promoter DNA and a tether-helix that interacts with TFAM in the initiation complex (37). Biochemical studies show that NTE or tether-helix deletion in h-mtRNAP decreases promoter-specific transcription activity (43, 44). The y-mtRNAP contains a NTE, but its structure is not known. *In vivo* studies indicate that the deletion of N-terminal ∼185 aa does not affect transcription initiation but decreases expression of y-mtDNA genes and destabilizes the y-mtDNA (45). Biochemical studies show that the deletion of 100 and 270 aa from the N terminus of y-mtRNAP does not affect transcription initiation, but deletion of 380 aa, the entire NTE region, affects promoter melting and transcription initiation (46). Thus, NTE has multiple roles that remain underexplored.

### Initiation factors—yeast MTF1 and human TFB2M

**Figure 3:**
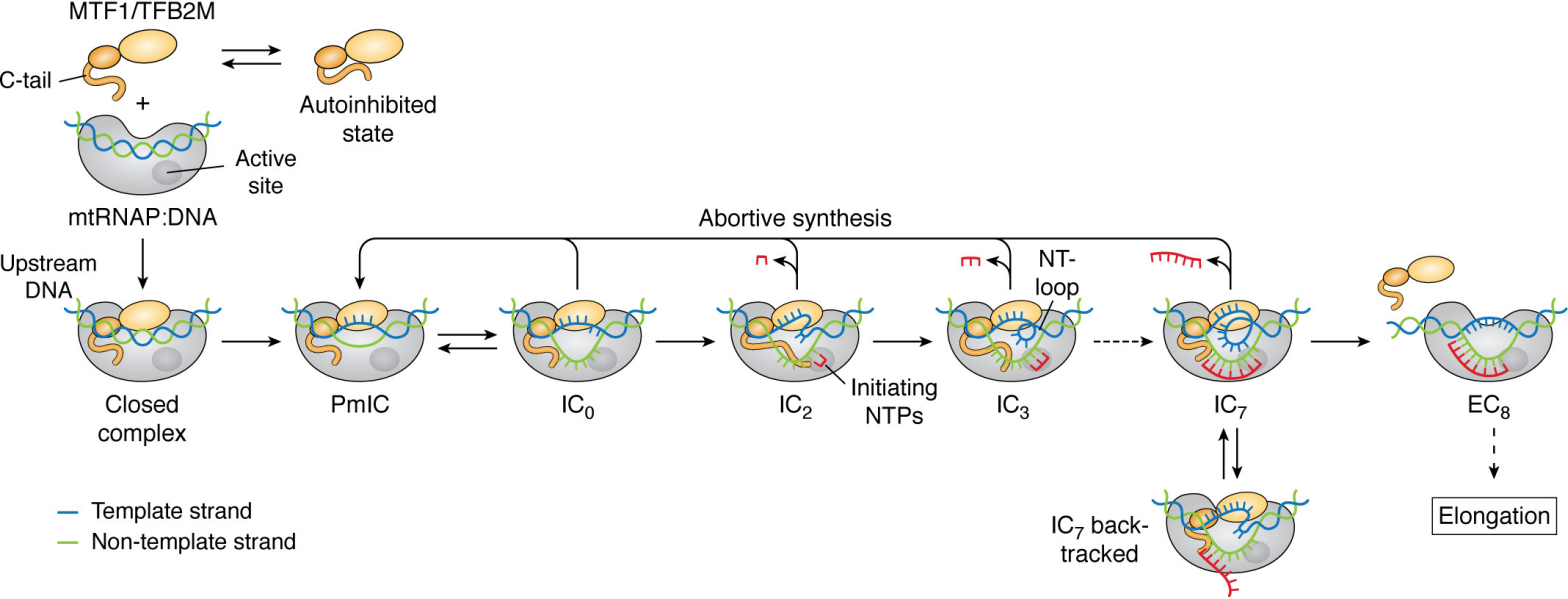
**Transcription initiation pathway of mtRNAP.** Transcription initiation factor MTF1/TFB2M (in *orange*) equilibrates between two states: an autoinhibited state, where the flexible C-tail occludes the DNA-binding site, and a free state, where the C-tail is free to interact with the mtRNAP. The exact pathway of closed complex formation is not known. Here, MTF1/TFB2M is shown to associate with a DNA-bound mtRNAP (in *gray*) to form a closed complex, in which DNA is slightly bent but not melted (template in *blue*, nontemplate in *green*). In the h-mtRNAP complex, TFAM (not shown here) would be bound to the upstream DNA assisting in promoter-specific binding. Studies of y-mtRNAP indicate an intermediate, PmIC, between the closed complex and initiation complex. In the PmIC state, base pairs from position −4 to −1 melt, MTF1/TFB2M stabilizes the bubble by interacting with the nontemplate strand, and mtRNAP interacts with the template strand. Subsequently, +1 and +2 base pairs melt to generate an IC_0_ state. The MTF1/TFB2M C-tail helps position the template strand in the active site to promote binding +1 and +2 initiating NTPs in the IC_2_ state. The binding of the initiating NTPs drives the conversion of PmIC to IC_2_. Phosphodiester bond formation results in a 2-bp RNA:DNA hybrid, which elongates in a stepwise manner through melting of the downstream DNA and scrunching of the nontemplate strand into an NT-loop, as shown in the IC_3_. The growing RNA:DNA hybrid and the NT-loop push the C-tail away from the active site cavity and help the transition into elongation after 8-nt RNA synthesis. During the transition into elongation, the promoter DNA unscrunches and unbends, and the −4 to −1 base pairs of the bubble reanneal. MTF1/TFB2M may entirely or partially dissociate during the transition into elongation. Branched pathways occur with some frequency during transcription initiation, resulting in abortive synthesis or backtracking of the mtRNAP. During abortive synthesis, the RNA transcripts in IC_2_ to IC_7_ dissociate into the solution; the mtRNAP remains bound to the promoter DNA in the PmIC/IC_0_ state and rebinds NTPs, starting another round of transcription reaction. During backtracking, the RNA does not dissociate, but downstream DNA reanneals, fraying the 3′-end of the RNA:DNA hybrid.

**Figure 4:**
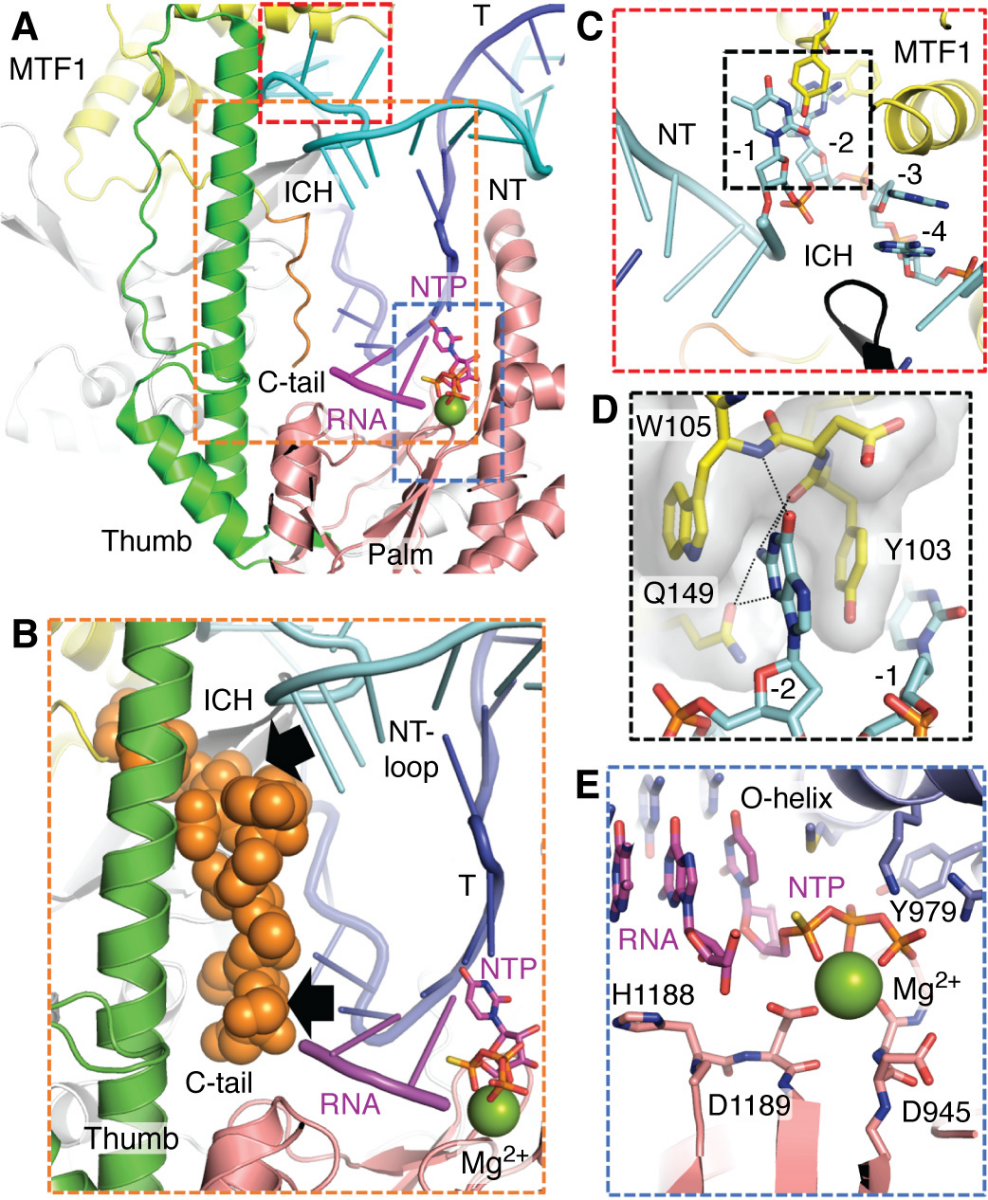
**Detailed views of the active-site cavity in the initiation complexes of yeast mtRNAP.***A*, view into the active-site cavity of the IC_2_+NTP complex showing the scrunched nontemplate strand (*cyan*), the melted template strand (*blue*) aligned with the 2-mer RNA (*magenta*), the next incoming NTP (*red*), and the catalytic metal ion (*green*). The conserved elements, including the thumb domain (*green*), ICH (*gray*), MTF1 C-tail (*orange*), and palm domain (*salmon pink*), are stabilizing the melted template and nontemplate DNA strands in the active site. *B*, the MTF1 C-tail is highlighted to show its proximity to the 5′-end of the RNA:DNA hybrid and the scrunched NT-loop. The C-tail is expected to sterically clash (*black arrows*) with the RNA:DNA and NT-loop. *C*, the partially melted initiation complex (*PmIC*) shows the flipped −4 to −1 bases of the melted nontemplate strand interacting with the ICH of y-mtRNAP and MTF1. *D*, a detailed view of the base-stacking and base-specific interactions of the −2 G of the nontemplate strand with the residues of MTF1. *E*, an in-depth look of the active site of IC_2_+NTP with 2-mer RNA and incoming NTP interactions with the fingers (O-helix) and palm domain residues. The Mg^2+^ (*green*) in the structure is coordinated with the NTP and residues of the palm domain.

TFB2M and MTF1 are both essential transcription factors of h-mtRNAP and y-mtRNAP, respectively. TFB2M was discovered based on its amino acid sequence homology to MTF1 (47, 48) and after MTF1 was established as the initiation factor in yeast (49, 50). In coordination with the respective mtRNAP subunits, these initiation factors recognize and melt the −4 to +2 region of the promoter DNA to bring about promoter-specific transcription initiation. MTF1 and TFB2M are evolutionarily related to rRNA methyltransferases (37, 51). However, they have lost most of their ancestral methyltransferase activity while retaining their nucleic acid–binding function (52, 53). TFB2M and MTF1 are dumbbell-shaped with a large NTD that contains a nucleic acid–binding groove that interacts with the nontemplate strand of the initiation bubble, and a smaller CTD with a flexible C-terminal tail (aa 320–340 in MTF1 and 380–396 in TFB2M) (6, 37) (Figs. 2*C* and 4). The structure of free TFB2M resolved the C-tail folded within the nucleic acid–binding groove of the CTD (37). Biochemical studies show that the C-tail autoinhibits the DNA-binding activity of MTF1 and TFB2M, preventing the free factors from associating tightly with the DNA. The C-tail's deletion enabled these factors to bind DNA with a higher affinity (52) (Fig. 3). The C-tail undergoes a conformational change and begins to play an active role in transcription initiation when these factors bind to their respective mtRNAP subunit partners (52), as discussed below.

### Initiation factor—human TFAM

TFAM is abundantly present in human mitochondria, in amounts sufficient to coat the mtDNA, and it plays essential roles in mtDNA packaging and nucleoid formation (54, 55, 56). The yeast ABF2 is a TFAM homolog with similar roles in the yeast mitochondria. Both proteins contain two high-mobility group (HMG) box domains that bind ∼30 bp of DNA while bending the DNA into a U-turn conformation that facilitates DNA packaging (57, 58, 59, 60). Additionally, cross-strand binding of TFAM is observed and likely necessary for nucleoid formation (61). Unlike ABF2, which is needed only for the packaging and maintenance of y-mtDNA, TFAM has an additional role in transcription initiation (62). TFAM contains a characteristic C-terminal tail, which is missing in ABF2, which enables TFAM to moonlight as a transcription factor (62, 63). The C-tail of TFAM is needed for optimal DNA bending (64), dimer formation (65), DNA loop formation (20), and interactions with h-mtRNAP (37). The C-tail in TFAM activates LSP transcription and is essential for HSP1 transcription (20). A structural study showed that TFAM binds in two distinct orientations on HSP1 and LSP DNA fragments, placing the C-tail of TFAM in opposite directions to h-mtRNAP at the transcription initiation site (66). A protein-protein cross-linking study suggested that the opposite orientation of TFAM on HSP1 was due to missing interactions in the short DNA fragment used in the structural studies (67). Whereas structures of initiation complexes show that TFAM is oriented in the same way on LSP and HSP1 (37), it is possible that TFAM on its own binds in two different orientations on HSP1 while having a specific orientation on LSP. With multiple roles in mtDNA transcription, maintenance, and biogenesis, TFAM is undoubtedly a key protein in human mitochondria that needs to be better understood. Additionally, TFAM needs to be regulated and maintained at appropriate levels for healthy mitochondrial functions (68).

## Mechanisms of transcription initiation

### Assembly of the transcriptional complex and promoter binding

Transcription of the mtDNA initiates after mtRNAP, and the transcription factors assemble at the promoter site to form a closed complex (Fig. 3). There is no existing structure of a closed complex of mtRNAP; however, recent single-molecule studies of y-mtRNAP suggest that the the DNA in the closed complex is slightly bent, and the closed complex is in equilibrium with the open complex (69). We have a limited understanding of the assembly mechanism of h-mtRNAP and transcription factors at each of the three h-mtDNA promoter sites. h-mtRNAP and TFB2M cannot recognize the promoter sequences on their own. However, footprinting studies show that TFAM can specifically bind to LSP (20, 62, 70), and cross-linking studies show that TFAM and h-mtRNAP can form a complex on the DNA (43). Based on these studies, the current literature model suggests that TFAM recruits h-mtRNAP to the promoter site, where the two proteins form a pre-initiation complex. TFB2M then binds to the pre-initiation complex to generate the open initiation complex. This model is based mostly on LSP studies, and the assembly mechanism on HSP1 could be different because a high-affinity TFAM site is not apparent in footprinting studies of HSP1 (20, 62, 70). Moreover, it remains to be determined how TFAM specifically recruits h-mtRNAP to the promoter site *in vivo,* given that TFAM is bound everywhere on h-mtDNA and TFAM:h-mtRNAP complex formation is not promoter-specific (43). Another mechanism of assembly that is not mutually exclusive with the current model is that h-mtRNAP and TFB2M complex binds to the promoter and recruits TFAM. Studies show that h-mtRNAP and TFB2M can bind DNA on their own with a high affinity (*K_d_* values of 2 and 50 nm, respectively) (27, 52, 64), and the two proteins can form a specific complex on promoter DNA (44) and catalyze promoter-specific transcription, albeit at low levels (26, 27). A recent study suggested that TFAM-mediated DNA looping is involved in the assembly and activation of the transcriptional complex on HSP1 (20). Further studies are needed to better understand how the initiation complex is generated at each of the three promoters, at each of three h-mtDNA promoters, a critical first step that controls gene expression and mtDNA replication.

The initiation complex structure with h-mtRNAP, TFAM, and TFB2M shows that the three proteins cover the promoter DNA from position −40 to +8 (37). TFAM contacts the −40 to −16 region, the PPR domain of h-mtRNAP interacts with the −15 to −12 region, and the specificity loop of h-mtRNAP contacts base pairs around the −7 region (Fig. 2*C*). TFB2M interacts with the promoter region from position −8 to +2, which includes the initiation bubble region. The fingers and palm domain of the CTD in h-mtRNAP contact the downstream DNA. TFB2M directly interacts with h-mtRNAP in the initiation complex; the CTD of TFB2M interacts with the TFB2M-hairpin in h-mtRNAP (37).

The two components of the yeast transcription initiation complex do not recognize the promoter sequence independently as the human system. The y-mtRNAP binds both promoter and nonpromoter sequences with similar affinities (∼60 nm *K_d_*) (71). MTF1, on the other hand, has a very weak affinity for DNA because its C-tail almost completely autoinhibits its DNA-binding activity (Fig. 3) (52). When y-mtRNAP and MTF1 form a complex, they bind DNA with a ∼300-fold higher affinity relative to y-mtRNAP alone. The y-mtRNAP:MTF1 complex recognizes the promoter and bends the DNA sharply around the initiation site (71). Promoter bending is necessary for generating a productive initiation complex (Fig. 2*C*) and is a conserved feature observed in all single-subunit RNAPs (35, 36, 37).

Footprinting studies show that y-mtRNAP protects ∼30 bp of promoter DNA from approximately −15 to +15 (72). Unlike h-mtRNAP, the y-mtRNAP lacks extensive contacts with the upstream promoter region, conferred by TFAM and the NTE regions in h-mtRNAP. The recent cryo-EM structures of y-mtRNAP and MTF1 show base-specific interactions of MTF1 with the conserved nonanucleotide promoter sequence from position −8 to +1 (Fig. 4, *C* and *D*) (36). The structure did not resolve the NTE in y-mtRNAP; hence, it is not clear whether y-mtRNAP has the equivalent PPR domains present in h-mtRNAP. Like T7 RNAP, the y-mtRNAP contains a prominent AT-rich recognition loop, which interacts with the −16 region. In h-mtRNAP, the AT-rich loop is much smaller, and it makes minimal contacts with the promoter. Interestingly, y-mtRNAP contains a unique ∼90-aa insertion in its CTD, absent in h-mtRNAP (Fig. 2*B*). The CTD insertion in y-mtRNAP interacts with the downstream DNA around +11 bp. The CTD insertion interactions stabilize the bent conformation of the DNA around the initiation site, whereas MTF1 stabilizes the DNA at the opposite end (36). Perhaps due to these additional promoter contacts with downstream DNA, y-mtRNAP does not need ABF2, the TFAM homolog. Loss of downstream DNA interactions in h-mtRNAP and dependence on TFAM may have coevolved for regulation.

MTF1 shows several contact points with the y-mtRNAP; the CTD of MTF1 interacts with a prominent hairpin in y-mtRNAP adjacent to the intercalating hairpin (MTF1-hairpin equivalent to the TFB2M-loop in h-mtRNAP), and the NTD of MTF1 contacts the thumb domain of y-mtRNAP (36, 73). The thumb domain in h-mtRNAP is projected toward TFB2M, suggesting interactions like in y-mtRNAP (Fig. 2*C*), but the crystal structure shows a disordered thumb-tip in the h-mtRNAP (37). Comparison of h-mtRNAP and y-mtRNAP initiation complexes shows a conserved active-site cleft, a similar trajectory of the bent upstream and downstream DNA arms, and similarly positioned TFB2M and MTF1 bound to the respective mtRNAP subunits. Overall, the architecture of h-mtRNAP and y-mtRNAP on the promoter DNA and many of the interactions are highly conserved. Hence, the recent cryo-EM structures of two transcription initiation states of y-mtRNAP are excellent models for understanding the underlying multistep mechanism of transcription initiation (Fig. 3).

### Promoter melting

DNA bending and untwisting converts the closed complex to an open complex in which base pairs from −4 to +2 with respect to the transcription start site at +1 are melted (Fig. 3). All of the single-subunit RNAPs studied thus far contain a similarly sized initiation bubble at a common location on the promoter DNA (27, 34, 35, 37, 74, 75, 76, 77). T7 RNAP generates this initiation bubble on its own, whereas h-mtRNAP and y-mtRNAP rely on TFB2M and MTF1, respectively, to create the initiation bubble. Fluorescence 2-aminopurine promoter melting studies indicate that the optimal melting of LSP DNA from −4 to +2 requires both TFB2M and TFAM, and initiating NTPs enhance the melting of the −1 to +3 base pairs (27). The intercalating hairpin is a conserved element in all single-subunit RNAPs that plays a crucial role in promoter melting by acting as a wedge and preventing initiation bubble collapse (78). Once the promoter is melted, the template strand around the transcription start site is held by the mtRNAP subunit, and the nontemplate strand is trapped by MTF1/TFB2M (36, 79, 80). Base-specific interactions are involved in initiation DNA bubble formation in T7 RNAP and y-mtRNAP, which is probably the case in h-mtRNAP; *in vitro* transcription studies show that mutations in the −4 to −1 base pairs in LSP decrease runoff RNA synthesis (81). Additional structural studies are needed to understand the role of base-specific interactions in the mechanism of promoter melting by h-mtRNAP.

Structural and single-molecule FRET studies of y-mtRNAP show that closed complex to open complex formation occurs in two steps. There is an intermediate state between closed DNA and the fully open DNA bubble (36, 69) (Fig. 3). Cryo-EM studies captured this new intermediate's high-resolution structure, showing that the state has a partially melted initiation bubble from −4 to −1 (36). This newly identified partially melted initiation complex (PmIC) has a shallower DNA-bending angle of ∼120° between the upstream and downstream arms of the promoter DNA compared with a sharper ∼60° bend between two arms in the fully melted initiation complex. MTF1 plays a vital role in forming the PmIC as it makes base-specific interactions with the flipped −3 and −2 bases of the nontemplate strand, and biochemical studies show that MTF1 also facilitates DNA bending (36, 71, 74) (Fig. 4, *C* and *D*). The base-specific interactions of MTF1 with the promoter are consistent with the existing promoter DNA mutational studies (82, 83). The transcription start site in the PmIC is duplexed; hence, PmIC is not catalytically active, and the +1 and +2 base pairs must be melted to convert PmIC to a catalytically active IC_0_ state (Fig. 3). An IC_0_ state structure has not been determined; however, cryo-EM studies have determined the structure of the IC_2_+NTP intermediate with a 2-mer RNA and an incoming NTP, which provides insights into the change from PmIC to IC (36). Multiple large-scale conformational changes accompany the transition from PmIC to the IC in y-mtRNAP, including further DNA bending, expansion of the initiation bubble, template strand alignment in the active site, and new interactions of the CTD insertion region with the downstream DNA. The template strand of the initiation bubble undergoes a substantial conformational change in the transition from PmIC to IC as it positions near the active site with the RNA and NTP. The MTF1 C-tail plays a crucial role by stabilizing the template strand in the active site (36, 84). The active-site cavity accommodates the expanded transcription bubble by scrunching the nontemplate strand of the promoter DNA into a loop, as we discuss below.

Several studies have suggested that the +1 and +2 base pairs' identity dictates the efficiency of transcription initiation (85, 86), and this sensitivity is the basis for the ATP-sensing mechanism in the yeast mitochondria. Cryo-EM studies of y-mtRNAP show that PmIC to IC conversion is driven by initiating NTP binding, suggesting a crucial role of PmIC to IC transition in the ATP-sensing mechanism. Whether such a mechanism exists in human mitochondria remains to be determined. Overall, the promoter melting mechanism is likely to be more complex in h-mtRNAP with the involvement of both TFB2M and TFAM. It will be interesting to determine whether a PmIC-like complex exists in the h-mtRNAP pathway and whether the general mechanism of promoter melting and template strand alignment are conserved between the yeast and human systems. The studies of y-mtRNAP have provided a basic framework for future studies of the h-mtRNAP system.

### Transcription initiation

Transcription is initiated with the synthesis of 2-mer pppNpN RNA from NTP molecules base-paired to the +1 and +2 templating positions at the start site (Fig. 3). The flexible C-tail in MTF1/TFB2M facilitates the binding of initiating NTPs to bring about the optimal synthesis of 2-mer RNA (84). Structural studies show that a specific element in mtRNAPs called the TFB2M/MTF1-hairpin guides the C-tail toward the active site cavity, where the C-tail is stabilized by the intercalating hairpin and the thumb domain of the mtRNAPs (37). Structural studies resolved a partial structure of the TFB2M C-tail in the h-mtRNAP IC state, whereas the y-mtRNAP IC_2_+NTP state resolved the entire MTF1 C-tail structure in the active site cavity (Fig. 4, *A* and *B*) (36). Interestingly, the MTF1 C-tail interacts with both the template and nontemplate strands of the initiation bubble near the transcription start site, explaining its role in transcription initiation. The MTF1 C-tail interactions with the template are consistent with protein-DNA cross-linking studies that indicated the proximity of MTF1 C-tail to the −3/−4 template base and the nontemplate strand (87, 88). Interestingly, the C-terminal amino acids in the C-tail approach close to the 5′-end of the 2-bp RNA:DNA hybrid, which suggests that the C-tail will sterically clash with the elongating RNA:DNA hybrid during transcription initiation (36), and this is consistent with biochemical studies (84). Any clashes between the C-tail of TFB2M and the RNA:DNA hybrid in h-mtRNAP remain to be determined. The steric clashes produce abortive products but play an essential role in the transition into elongation, as discussed below.

### RNA synthesis

The active-site structural features are conserved among T7 RNAP, y-mtRNAP, and h-mtRNAP (Fig. 2*C*). The fingers domain is a mobile region near the active site involved in RNA synthesis (35). The y-mtRNAP structure in the IC_2_+NTP state has captured the fingers domain in a catalytically active state (36). The finger domain in the h-mtRNAP IC structure is caught in a different rotational state referred to as the clenched conformation that does not support template or NTP binding (37). There are two conserved helices in the fingers domain, the O-helix and Y-helix, which are essential for RNA synthesis. In the rotated clenched conformation, the Y-helix is sterically hindering the template/NTP from binding into the active site. Whether the clenched state is an artifact of crystallization or a branched state in the initiation pathway remains to be determined. The inactive clenched conformation must adopt a conformation as in y-mtRNAP for 2-mer synthesis (36). In the active state, the O-helix shows extensive interactions with the incoming NTP. A conserved tyrosine in the O-helix interacts with the 2′-OH of the incoming NTP and provides specificity for binding rNTPs over dNTPs. The active site utilizes a two-metal–dependent reaction mechanism for nucleic acid polymerization (89, 90). The y-mtRNAP IC structure shows only one catalytic Mg^2+^ ions at the active site chelating the catalytic residues in the palm domain and the phosphate groups of the incoming NTP (Fig. 4*E*). The second metal ion, which was not observed in this structure, would be bound close to the 3′-OH of the 2-mer RNA. The Y-helix in the fingers domain wedges against the downstream junction of the initiation bubble, and this conformation is consistent with its role in aiding downstream DNA strand separation for RNA elongation, as observed in the elongation complex structures of T7 RNAP and h-mtRNAP (39, 91).

### DNA scrunching and abortive synthesis

During transcription initiation, the RNA gets elongated from 2 nt to ∼8–10 nt in length. Throughout this process, the RNAP remains stably bound to the promoter DNA. Due to these stable promoter interactions, the RNAP cannot translocate downstream to elongate the RNA transcript as it does during the elongation phase. Instead, the newly melted template and nontemplate strands are brought into the active-site cavity to guide RNA transcript synthesis. The template strand directs the synthesis of RNA and remains base-paired to the nascent RNA, forming an RNA:DNA hybrid, whereas the nontemplate strand (NT) remains single-stranded but gets scrunched into an NT-loop (Fig. 4*B*). Fluorescence studies have provided evidence for DNA scrunching in both single-subunit and multisubunit RNAPs (35, 92, 93). Recently DNA scrunching was demonstrated in y-mtRNAP by single-molecule FRET and cryo-EM studies (36, 69, 84). However, the cryo-EM structure of y-mtRNAP IC_2_+NTP captured the scrunched DNA conformation for the first time and showed that scrunching generates an NT-loop (36). The NT-loop is stabilized in the active-site cavity by the MTF1 C-tail, y-mtRNAP thumb, and intercalating hairpin. Stabilization of the NT-loop by the MTF1 C-tail is consistent with single-molecule FRET studies that showed that C-tail deletion decreases DNA scrunching (84). As RNA synthesis continues during transcription initiation and generates IC_2_ to IC_7_ intermediates, we expect the NT-loop to grow in size, as shown in Fig. 3.

Cryo-EM studies of y-mtRNAP show that the IC_2_+NTP has a scrunched DNA conformation, whereas the DNA in the PmIC state is not scrunched. PmIC and IC states' coexistence in the cryo-EM experiment suggests a constant switching between the two states through dissociation and rebinding of 2-mer RNA and NTP (69). Dissociation of short RNA transcripts is observed in all DNA-dependent RNAPs during transcription initiation in a process termed abortive synthesis (Fig. 3). Although abortive synthesis's mechanism and significance are not entirely understood, DNA scrunching-unscrunching transitions have been implicated in RNA transcript dissociation (35, 69, 92, 93, 94, 95). The coexistence of IC_2_+NTP and PmIC states suggest that after RNA transcripts dissociate into solution, the initiation complexes revert to the PmIC state, which then equilibrates to the IC_0_ state to restart another cycle of transcription initiation by binding new molecules of NTPs, as shown in Fig. 3. Steric clashes of the RNA:DNA hybrid with specific elements in the RNAP represent a possible mechanism for abortive synthesis (96, 97, 98). In y-mtRNAP, the MTF1 C-tail buttresses against the 5′-end of the 2-mer RNA:DNA hybrid and the NT-loop (Figure 3, Figure 4 (*A* and *B*)); hence, the C-tail is in a position to clash with the growing RNA:DNA hybrid and NT-loop to trigger abortive synthesis. Biochemical studies showed reduced abortive synthesis upon MTF1 C-tail deletion, consistent with this model (84). The element analogous to the C-tail in multisubunit RNAPs is the 3.2 finger of bacterial σ-factor and B-reader of eukaryotic TFIIB initiation factor; 3.2 finger deletion mutants have similar effects of reducing abortive synthesis (96, 99, 100).

Single-molecule studies of y-mtRNAP also detected another branched pathway where DNA unscrunching occurred without RNA dissociation (69). These RNA-bound unscrunched complexes were proposed to be backtracked complexes that result from fraying of an uncertain number of base pairs from the 3′-end of the RNA:DNA hybrid, as shown in Fig. 3. Branched pathways can control the efficiency of transcription initiation and hence are potential targets for transcription regulation. There is extensive evidence that backtracked complexes play essential roles in transcription regulation in multisubunit RNAPs (101). It remains to be determined whether backtracking occurs in h-mtRNAP and has similar roles in regulating transcription.

### Transition from initiation to elongation

When the RNA:DNA hybrid reaches a length of 8–10-bp, several conformational changes occur that transform the initiation complex into an elongation complex (Fig. 3). Transition into elongation is a critical barrier for productive RNA transcript synthesis, and promoter release is a key step that triggers this event. In addition to promoter release, other conformational changes must occur to make a stable elongation complex, including unbending and repositioning the upstream DNA, the initiation bubble's collapse, and the release of the initiation factors. These events are better-characterized in T7 RNAP (91, 94, 102, 103), and our understanding of this process in mtRNAP is limited to a few studies of h-mtRNAP and y-mtRNAP. Comparison of the structures of initiation and elongation complexes of T7 RNAP and h-mtRNAP shows that the downstream DNA remains stably bound to the RNAP when the initiation complex changes into an elongation complex. Most changes occur at the upstream DNA, with loss of promoter contacts and promoter unbending (34, 35, 37, 39, 104). Single-molecule FRET studies of y-mtRNAP detected promoter unbending as an abrupt and irreversible change after 8-nt synthesis (69), suggesting that the transition into elongation commences in y-mtRNAP when the RNA:DNA hybrid reaches a length of 8 bp. Next, the initiation bubble collapses with the reannealing of the initially melted −4 to −1 bases; this process occurs gradually between 8- and ∼10-nt RNA synthesis (84). Bubble collapse is necessary to create a single-stranded RNA transcript, which gets threaded into the RNA-exit channel to generate a stable elongation complex.

## Mitochondrial transcription elongation and termination

### Dissociation of initiation factors and transcription elongation

Displacement of the initiation factors from mtRNAP must occur during the transition into elongation; however, the timing of initiation factor dissociation has not been resolved. Pulldown assays suggest that MTF1 dissociates from y-mtRNAP when the RNA transcript reaches a length of ∼13 nt (105). Complete dissociation of the initiation factor is not obligatory; the factor could remain bound at an alternative site and continue to regulate transcription elongation. The CTD of TFB2M disengages from the TFB2M-hairpin during the transition from initiation to elongation (38, 39). Studies of y-mtRNAP indicate that the MTF1 C-tail's steric clashes with the RNA:DNA and NT-loop are involved in this process (84). Whether the C-tail of TFB2M has similar roles as the C-tail of MTF1 in promoting transition into elongation remains to be determined. TEFM has been identified in human mitochondria as an elongation factor, and structural studies show that TEFM binds to the nontemplate strand of the transcription bubble (79, 106). This conformation suggests that TFB2M is released after TEFM takes its place on the elongation complex's transcription bubble. It is also not known whether h-mtRNAP dissociates from TFAM during the transition into elongation. For a better understanding of the mechanism of transition from initiation to elongation, it will be necessary to use real-time and direct monitoring of the association and dissociation of the initiation and elongation factors during transcription.

*In vitro* transcription studies show that y-mtRNAP and h-mtRNAP are active in catalyzing transcription elongation on a premade RNA:DNA hybrid (79, 106, 107). Kinetic studies show that h-mtRNAP elongates the RNA and adds a correct nucleotide at a rate of ∼10 nt/s, showing an error rate of 2 × 10^−5^ incorrect nucleotide addition over a correct nucleotide addition (108). Additionally, h-mtRNAP can synthesize ∼500-nt-long RNA transcripts; however, h-mtRNAP requires TEFM to make longer ∼4000-nt-sized transcripts (79, 106, 107). Structural studies show that TEFM interacts with the transcription bubble and possibly RNA to stabilize the elongation complex (79). Biochemical studies show that TEFM stabilizes the elongation complex by decreasing the off-rate of h-mtRNAP by ∼60-fold (108). *In vitro* transcription studies show that h-mtRNAP frequently pauses on normal templates and terminates on G-rich sequences, including G-rich sequences downstream of LSP, and these events reduce in the presence of TEFM (107, 109, 110). TEFM also stimulates mutagenic bypass over 3′-end mismatches (108) and 8-oxo-dG lesions (107). The transcripts terminated at the G-rich sequences downstream of LSP in h-mtDNA, and downstream of replication promoters of y-mtDNA, are proposed to prime mtDNA replication (111, 112, 113, 114, 115). Still, the exact mechanism of replication initiation is not understood. PRO-Seq studies of mtDNA in living cells have located transcription-pausing sites just upstream of the position where transcription to replication transition occurs on both light and heavy strands, which may be necessary for initiating mtDNA replication (25). TEFM, with its ability to decrease transcription termination and pausing, has been implicated in regulating mtDNA replication (109). Recent studies show that reduced TEFM levels do not reduce mtDNA synthesis (116). Thus, the role of TEFM in h-mtDNA replication regulation requires further investigation.

The y-mtRNAP catalyzes RNA synthesis on premade RNA:DNA substrates with a 5-fold faster rate of ∼50 nt/s relative to h-mtRNAP and shows a lower error rate of 6 × 10^−6^ incorrect nucleotide addition over a correct nucleotide addition (108). The most frequent error observed *in vitro* by y-mtRNAP and h-mtRNAP is A to G substitution in RNA. The y-mtRNAP can synthesize an RNA primer on ssDNA at 3′-purine(pyrimidine)_2-3_ sequences, and these RNA primers are elongated to kilobase-sized RNA products (41). Thus, it is possible that y-mtRNAP, like T7 RNAP, does not need an elongation factor to catalyze processive RNA elongation. A few studies have suggested that the DEAD-box protein Mss116p may serve as the elongation factor of y-mtRNAP (117, 118). However, additional studies are needed to investigate how Mss116p facilitates transcription elongation by y-mtRNAP.

### Transcription termination

In humans, transcription from LSP and HSP promoters generates a polycistronic RNA transcript, processed to make the individual mRNA, tRNA, and rRNA molecules. Sequencing of h-mtDNA nascent transcripts has suggested that light-strand transcription terminates between positions 2612 and 3252, and heavy-strand transcription terminates within the D-loop between positions 16,076 and 195 (25). Another study suggested that heavy-strand transcription ends near the core termination-associated sequence site (around position 16,090 in h-mtDNA) (119). MTERF1 was identified as the transcription termination factor in human mitochondria in 1989 (120). MTERF1 binds to h-mtDNA within the Leu-tRNA's coding region between positions 3232 and 3253 downstream of the rRNA genes transcribed from HSP1 (121, 122, 123, 124). Studies indicate that MTERF1 blocks transcription originating from the LSP more efficiently than from HSP1 (122, 125), suggesting that the termination mechanism of MTERF1 has a polarity, which prevents LSP transcription from generating antisense rRNAs. MTERF1 also pauses the mitochondrial helicase TWINKLE and the h-mtDNA replisome (126), but its significance is not understood.

The crystal structure shows that MTERF1 binds asymmetrically to the 22-bp termination sequence and distorts the DNA by unstacking three specific bases, including A3243 of the light strand and T3243 and C3242 of the heavy strand (124). The unstacking of the base pairs by MTERF1 is essential for the DNA complex's stability and transcription termination. The polar arrest by MTERF1 is analogous to the mousetrap mechanism of replication arrest by bacterial Tus-Ter complex, which also involves base unstacking (127, 128). Mutations in the MTERF1 DNA-binding sequence lead to a spectrum of diseases under the syndrome MELAS (mitochondrial myopathy, encephalomyopathy, lactic acidosis, and stroke-like episodes) with devastating neuromuscular consequences. Interestingly, *Mterf1* knockout mice display no overt phenotypes or respiration defects (123, 129). Mutation A3243G of one of these unstacked bases is associated with the mitochondrial disorder MELAS (130); however, it appears to be due to alteration of the Leu-tRNA structure (131). The connections between the MELAS-associated mutations and MTERF1 function have been clearly shown only *in vitro* (120).

Most promoters of the y-mtDNA make multigene RNA transcripts that are terminated and processed (132). However, proteins homologous to MTERF1 are not found in the yeast mitochondria, and the mechanism of transcription termination on y-mtDNA is likely distinct from that on h-mtDNA.

## Regulation of mitochondrial transcription

**Figure 5:**
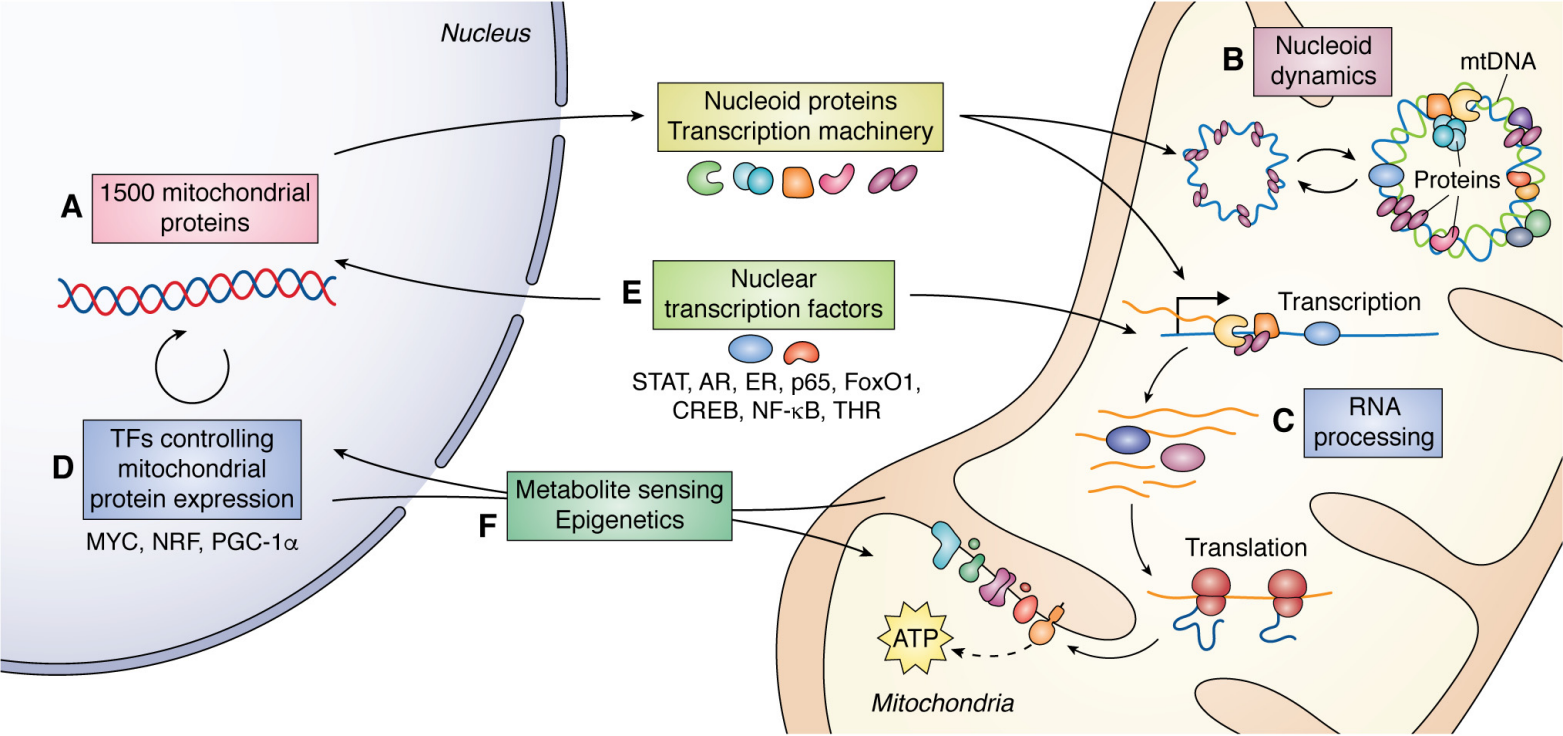
**Regulation of mitochondrial transcription overview.***A*, most mitochondrial proteins, including the core mtDNA transcription machinery and other nucleoid proteins, are encoded by nuclear genes, synthesized by cytosolic ribosomes, and imported into the mitochondria. Therefore, mitochondrial transcription and its regulation are largely dependent upon nuclear-encoded factors. *B*, a subset of these proteins associate with mtDNA, forming nucleoid particles. Nucleoid dynamics, including epigenetic modifications to mtDNA, nucleoid protein interactions, and post-translational modifications of mtDNA transcription factors, affect mtDNA accessibility and transcription. *C*, other nuclear-encoded mitochondrial proteins are responsible for processing nascent mtRNAs. Prior to translation, polycistronic RNAs must be cleaved, chemically modified, and adenylated to reach their mature form. *D*, nuclear transcription factors regulate the expression of nuclear-encoded mitochondrial proteins like TFAM and h-mtRNAP, indirectly regulating the expression of mtDNA-encoded genes. *E*, additionally, various canonically nuclear transcription factors translocate to the mitochondria or shuttle between the two compartments under various conditions, providing one means of coordinating mitochondrial and nuclear gene expression. *F*, mitochondrial factors also influence nuclear epigenetics, contributing to retrograde signaling and cross-talk between the two compartments. Finally, both the nucleus and the mitochondria sense and respond to metabolic conditions, such as nutrient availability and reactive oxygen species patterns. These broad cellular states affect gene expression in both compartments and influence signaling between the two.

Having established the structural and mechanistic basis of transcription initiation, we now turn to recent insights into the primary modes of transcriptional regulation in mammalian systems (Fig. 5). We first discuss the direct modulation of the transcription machinery, including how accessory proteins, post-translational modifications, and DNA sequence and modifications regulate h-mtRNAP and initiation factors. We then highlight factors external to the mitochondria and feedback mechanisms between the nucleus and mitochondria that are implicated in transcriptional control.

### Direct control of the mitochondrial transcription initiation machinery

##### RNA polymerase

One of the most direct modes of transcriptional control in the mitochondria is through the modulation of the RNAP's access to mtDNA or its activity. H-mtRNAP is regulated by proteins that directly interact with mtDNA, termed nucleoid proteins. Nucleoid formation promotes mtDNA stability and may allow for genome regulation. Nearly 60 nucleoid proteins have been identified (133, 134) that fall into three main classes: the replication and transcription machinery, enzymes involved in metabolism, and quality control proteins. Nuclear DNA encodes all nucleoid proteins; therefore, mitochondrial transcriptional regulation is likely dependent upon the nucleus (Fig. 5, *path A*). Several nucleoid proteins, including mitochondrial ribosomal protein L12 (MRPL12), leucine-rich pentatricopeptide repeat–containing protein (LRPPRC), TEFM, and MTERF1, act as secondary factors that can control the function of h-mtRNAP, with some of these findings discussed above. Interestingly, homologous proteins in yeast playing similar roles have not yet been defined.

MRPL12 constitutes part of the large subunit of the mitochondrial ribosome; however, in its nonribosome-associated form, it interacts directly with h-mtRNAP and is thought to regulate h-mtRNAP's transcriptional activity (135). RNAi-mediated knockdown of MRPL12 destabilizes h-mtRNAP and decreases mtDNA transcription levels (136), suggesting that MRPL12 is necessary for h-mtRNAP stability. However, the necessity for MRPL12 in transcription is not seen in all studies (26), warranting further exploration of the function of MRPL12 in transcription, especially in light of the differential expression of MRPL12 observed in some cancers (137) and neurological disorders (138). LRPPRC is an additional protein thought to be a co-activator of mitochondrial transcription mediated through LRPPRC-h-mtRNAP complex formation and not through direct contact with mtDNA (139). Overexpression of LRPPRC in cell lines and mice results in increased mitochondrial transcripts (139, 140), whereas the loss of LRPPRC leads to decreased transcripts and mitochondrial dysfunction. Among mtDNA transcriptional machinery and regulators, LRPPRC is of great interest due to its direct implications in human diseases (recently reviewed in Ref. 141), including Leigh syndrome, a debilitating neurological disorder manifesting in the first year of a child's life. LRPPRC, as well as several other nucleoid proteins, also act in the processing of mitochondrial RNA in humans. Processing involves the cleavage of polycistronic transcripts, maturation of RNAs, and base modifications (Fig. 5, *path C*) and represents another form of mitochondrial transcription regulation (recently reviewed in Ref. 14).

##### Initiation factors

In mammalian systems, the initiation factors are essential for transcription; as such, regulation of TFAM and TFB2M represent another major mechanism of transcriptional control in mitochondria. TFAM is the most abundant protein component of mitochondrial nucleoids in mammals. TFAM coats and packages mtDNA, with higher TFAM:mtDNA ratios indicative of tighter packaging and reduced accessibility of the transcription and replication machinery (142) (Fig. 5, *path B*). However, TFAM binding to the promoter region is also needed for transcription initiation; therefore, altering levels of TFAM or the ability of TFAM to bind to mtDNA is a dominant mode of transcriptional control. Structural features of mtDNA, including G-quadruplex formation and mtDNA methylation, may impact TFAM binding.

The mitochondrial genome contains many potential G-quadruplex–forming sequences, especially on the guanine-enriched heavy strand (143). The potential roles for G-quadruplex structures in mitochondrial function were recently reviewed (144). *In vitro* studies show that TFAM binds G-quadruplex–forming DNAs with high affinity and structural specificity (145), but in culture ChIP-Seq experiments showed that TFAM avoids G-quadruplex sequences (146). Thus, further studies are needed to understand how G-quadruplex structures impact mammalian mtDNA transcription (147). Finally, there is an association of G-quadruplex–forming sequences with human mtDNA deletion breakpoints, highlighting these structures' biological and clinical importance (148).

Although still an area of some controversy, many reports indicate that mtDNA is subject to methylation, as evidenced by bisulfite sequencing, antibody-based approaches, and the presence of DNA methyltransferase activity within mitochondrial extracts, and this may be an important component of regulating mtDNA transcription (recently reviewed (149, 150)). Cytosine and adenosine methylation have been observed in the mitochondria (151, 152), albeit at low levels (153). Cytosine methylation has been identified at CpG (154, 155), GpC (156), and non-CpG loci (151, 157). One study observed the highest methylation frequency in the D-loop regulatory region (155), further supporting a role for methylation in controlling transcription and/or replication. GpC methylation induced by targeting a GpC methyltransferase to the mitochondria in a number of cell lines led to a decrease in mitochondrial transcripts, while leaving mtDNA copy number unchanged and not impacting cell function (156). Although the mechanisms remain unclear, such mtDNA modifications may be directly linked to the activity of mitochondrial transcription factors. The methylation of mtDNA alters TFAM binding and transcription activities, and may indirectly modulate TFB2M and h-mtRNAP through TFAM recruitment (158). Additionally, hypermethylation at the mtDNA D-loop impairs mtDNA transcription in cultured human cells (159). There is evidence that mtDNA methylation patterns are differentially regulated during development and aging (157), under hypoxic stress (152), and in vascular disease (159), supporting the functional relevance of these modifications. Finally, nuclear methyltransferases, such as DNMT1, DNMT3, and METTL4, have been shown to localize to mitochondria and methylate mtDNA, demonstrating the potential importance of mtDNA methylation in cross-talk between mitochondria and the nucleus (152, 157, 159).

In addition to DNA and protein structural regulation, post-translational modifications, including lysine acetylation and serine/threonine phosphorylation, serve as another layer of regulation of TFAM and TFB2M. Modification of TFAM affects DNA binding, compaction, transcription, and TFAM concentration levels (160, 161, 162, 163, 164). The phosphorylation of TFAM leads to degradation by the mitochondrial Lon protease, subsequently controlling the ratio of mtDNA and TFAM to regulate transcription (162, 165). Lon protease is one example of a nucleoid protein involved in mitochondrial quality control, along with a family of nucleoid proteins that includes ClpX protease and enzymes involved in the oxidative stress response (peroxiredoxin 5, methionine-*R*-sulfoxide reductase B2). These enzymes are centrally localized to protect the mitochondrial genome and preserve the replication and transcription machinery in times of stress, and additional studies are necessary to determine the importance of mtDNA proximity to their function. Like TFAM, TFB2M is also phosphorylated. Phosphorylation of TFB2M on two key threonine residues decreases the binding affinity of TFB2M for both mitochondrial promoters and its ability to carry out transcription *in vitro* (166). These phosphorylation sites are proposed to interact with the thumb domain of h-mtRNAP, thereby interfering with key interactions between TFB2M and h-mtRNAP and attenuating transcription initiation. Beyond the initiation factors, numerous post-translational modifications have been identified on h-mtRNAP (including phosphorylation, methylation, acetylation, and ubiquitination) and other proteins known to interact with mtDNA, but their roles in mediating changes in mitochondrial biology are largely unknown (167, 168, 169, 170). Additional support for the role of post-translational modifications in controlling mtDNA transcription is the mitochondrial localization of enzymes that catalyze both the addition and removal of these chemical marks, including a number of mitochondrial localized phosphatases and mitochondrial members of the sirtuin deacetylase family (SIRT3–5). Whereas a mitochondrial protein kinase has not yet been clearly identified, the histone acetyltransferase MOF and its regulatory partners KANSL1 and KANSL3 have been shown to localize to the mitochondria, bind to mtDNA, and regulate transcription. Genetic loss of MOF led to the accumulation of TFB2M and POLRMT on mtDNA when cells were cultured in galactose medium, a culture condition promoting the reliance on mitochondrial metabolism, potentially indicating stalled transcription (171). Future work to identify whether MOF is directly acetylating members of the transcriptional machinery to alter their function is required.

### Nuclear factors that control mitochondrial transcription

A major outstanding question is how the expression of nuclear and mitochondrial encoded genes is coordinated. Many nuclear proteins directly or indirectly impact mtDNA transcription; conversely, mitochondrial proteins, metabolites, and other factors influence nuclear gene expression. Some proteins play significant roles in both compartments and shuttle between the two. The diversity and interconnectedness of these cross-talk factors allow for nuanced molecular communication between the nucleus and the mitochondria, our understanding of which is still quite limited.

A critical aspect of regulating mtDNA transcription involves the nuclear transcription factors that control the expression of the mitochondrial transcription machinery (Fig. 5, *path D*). Nuclear respiratory factors 1 and 2 (NRF-1/2) and peroxisome proliferator–activated receptor coactivator 1α (PGC-1α) are widely established as “master regulators” of mitochondrial biogenesis and control the expression of many mitochondrial proteins, including h-mtRNAP (172), TFAM (173, 174), and TFB2M (175). NRF-1/2 and PGC1α play a role in the metabolic adaptation to changing nutrient status, partially through the modulation of mitochondrial function. NRF-2 also regulates the expression of MTERF1 (176), and decreased PGC-1α levels have been correlated with the down-regulation of MRPL12 (177) and LRPPRC (178), implicating these master regulators in the control of accessory transcriptional regulators as well as the core machinery. However, the extent to which these master factors directly control the accessory regulators' expression remains an open question and an intriguing area of future study.

In addition to nuclear transcription factors controlling nuclear gene expression, another mechanism of regulation involves localizing canonically nuclear transcription factors to the mitochondria, where they directly bind mtDNA and alter mtDNA transcription (Fig. 5, *path E*). Many such factors have been identified in previous studies and are discussed in prior reviews; these include thyroid hormone receptors, glucocorticoid receptors, cAMP-response element binding proteins, NF-κB subunits, myocyte enhancer factor 2 (MEF2D) (179), and signal transducer and activator of transcription (STAT) proteins (reviewed Refs. 11 and 180). Whereas recent findings have added several new nuclear transcription factors to the repertoire of those shown to localize to the mitochondria, in most cases, further investigation is needed to elucidate the direct roles of these transcription factors in controlling mitochondrial gene expression and to determine whether transcriptional responses are tissue- or context-dependent. Notably, a nuclear transcription factor in the mitochondria does not necessarily imply that it plays a role in regulating mtDNA transcription. Direct functional evidence must be obtained to support any claims of these factors' biological relevance in regulating mtDNA transcription (11, 181, 182). We must ensure that changes are not due to alterations in mtDNA copy number, translation efficiency, or mitochondrial integrity. As described in earlier sections of this review, structural and mechanistic studies have greatly aided our understanding of mtDNA transcription's basic process. Similar studies regarding nuclear transcription factors and other regulatory proteins within the mitochondria might illuminate how mtDNA transcription is controlled and coordinated with nuclear gene expression. Here, we report the most recent findings in this area, highlighting both new insights into factors previously known to localize to mitochondria (estrogen receptor, RelA, STAT3, FoxO3A) and nuclear transcription factors whose roles in the mitochondria have recently been discovered (androgen receptor, TEAD4, MDM2, FoxO1).

##### Androgen and estrogen receptors

The androgen receptor (AR) and estrogen receptors (ERα and ERβ) are ligand-dependent nuclear transcription factors that bind androgen and estrogen steroid hormones, respectively, and play diverse roles in cell function and development (183, 184). AR was recently found to localize to the mitochondrial matrix. AR appeared to negatively regulate the expression of several mitochondrial- and nuclear-encoded OXPHOS subunit genes in cultured prostate cancer cells. However, these effects may have occurred through altered TFAM expression and mtDNA content rather than through a direct role of AR in mtDNA transcription (185). Another recent study found that testosterone treatment of murine skeletal muscle cells increased TFAM expression, likely by up-regulating its nuclear expression regulators, NRF-1/2. Testosterone also increased the expression of mtDNA-encoded OXPHOS subunits, although this was proposed to occur indirectly through nuclear NRF/TFAM up-regulation. However, this study identified several putative AR-binding sites in mouse mtDNA by *in silico* analysis, indicating the possibility of direct regulation of mtDNA transcription by AR (186). Like AR, the estrogen receptors (ERα and ERβ) have been shown to localize to mitochondria and bind putative estrogen-response elements within mtDNA, and there is evidence that ERs may directly regulate the expression of both mitochondrial- and nuclear-encoded OXPHOS subunit genes (reviewed in Refs. 187 and 188). ERα and ERβ have been shown to indirectly up-regulate mtDNA-encoded OXPHOS subunits through the NRF/TFAM pathway (186, 188).

##### Transcription factor p65 (RelA) and STAT3

The NF-κB transcription factor family includes several transcription factors and their inhibitors, which play roles in signaling pathways involved in inflammation, cell differentiation, and cell survival (189). STAT proteins are another class of transcription factors involved in many signal cascades that regulate cell growth and survival (190). Mitochondrial localization was recently described for the proteins p50/NF-κB1 and RelA (p65), two subunits of the NF-κB family, as well as their inhibitor IκBα (11). However, another study found that only RelA and IκBα localized to mitochondria, and only RelA entered the matrix and associated with mtDNA (191). RelA recruitment to mtDNA decreased h-mtRNAP binding to mtDNA and the expression of two mtDNA-encoded OXPHOS subunit genes. This recruitment was not due to the direct binding of RelA to the mtDNA and was proposed to occur through an indirect mechanism (192). In mouse keratinocytes, STAT3 bound mtDNA and co-immunoprecipitated with TFAM. Furthermore, STAT3 knockout in these cells increased the transcription of several mtDNA-encoded genes, pointing to a potential inhibitory role of STAT3 in mtDNA transcription (193). However, STAT3 is known to play significant nontranscriptional roles in the mitochondria (194). Therefore, changes in mRNA levels of mtDNA-encoded OXPHOS genes in response to altered STAT3 should not be automatically interpreted as evidence that STAT3 directly impacts mtDNA transcription.

##### TEA domain transcription factor 4 (TEAD4)

The TEA domain family transcription factors (TEAD1–4) are downstream transcription factors of the Hippo signaling pathway and are essential in cell proliferation. A member of this family, TEAD4, is involved in energy homeostasis in early embryonic development (195, 196) and was recently shown to be implicated in mtDNA transcription control. In early embryonic cells, loss of TEAD4 led to decreased mitochondrial transcription and impaired OXPHOS, which was rescued by overexpression of an engineered TEAD4 containing a mitochondrial localization sequence (197). Using immunofluorescence and ChIP approaches, TEAD4 was shown to bind directly to mtDNA at the D-loop and other regions. Upon binding to mtDNA, TEAD4 interacts with h-mtRNAP on mtDNA and is proposed to facilitate transcription.

##### Mouse double-minute 2 proto-oncogene (MDM2)

MDM2 is well-known for its regulation of the nuclear transcription factor p53, an established tumor suppressor. However, MDM2 also has p53-independent functions, including binding to nuclear chromatin and altering transcription (198). Recently, MDM2 was shown to specifically bind the LSP, displacing TFAM and down-regulating transcription from the LSP in normal and cancer cells (199). Interestingly, the MDM2 effect appears to be promoter-specific, as numerous approaches indicate that MDM2 does not bind to the HSP or alter transcription from this promoter. The recruitment of MDM2 to the LSP reduces transcription of NADH dehydrogenase 6, thereby impairing Complex I activity. A rise in oxidative stress is a key factor driving MDM2 translocation to the mitochondrial matrix. This study added a new activity to the repertoire of oncogenic functions of MDM2 and provided an example of a mechanism underlying altered mitochondrial metabolism often found in tumor progression.

##### Forkhead box protein O1 and O3A (FoxO1, FoxO3A)

The four-member FoxO transcription factor family is comprised of nutrient-sensing proteins that control the transcription of nuclear genes involved in energy homeostasis, glucose metabolism, apoptosis, and cell cycle arrest. Recently, one member of this family, FoxO1, was shown to bind the mtDNA D-loop under basal metabolic conditions. Nutrient restriction in white and beige adipocytes led to FoxO1 shuttling from the mitochondria to the nucleus and binding to nuclear DNA, with a concomitant decrease in FoxO1 binding to mtDNA and transcription. This shuttling depended on specific nutrient restriction–induced reactive oxygen species patterns (200). The related transcription factor FoxO3A localizes to both the nuclear genome and mtDNA. Under low glucose, FoxO3A formed a complex with SIRT3, TFAM, and h-mtRNAP; this complex bound regulatory regions in mtDNA and increased mtDNA transcription (201). The shuttling of FoxO proteins between the nucleus and the mitochondria to regulate gene expression in response to reactive oxygen species and metabolic state points to intriguing avenues of communication between the mitochondria and the nucleus, an example of metabolite sensing mechanisms, as described below.

### Metabolite sensing in mito-nuclear coordination

The reliance of the mitochondria upon the nucleus is quite clear. Interestingly, many mitochondrial proteins have been shown to affect nuclear gene expression. Importantly, such retrograde signaling often depends on certain metabolic states or redox balance within the cell, highlighting how global cellular conditions function in interconnecting the nucleus and the mitochondria (Fig. 5, *path F*). In some cellular systems, TFAM was identified in nuclei and suggested to control nuclear gene expression (202, 203, 204). The mitochondrial PTEN-induced kinase PINK1 is implicated in retrograde signaling pathways to the nucleus in response to genotoxic stress. This signaling involves altered reactive oxygen species patterns and ATP production and triggers nuclear responses for maintaining cellular homeostasis (205). A unique form of retrograde mitochondrial signaling requires MOTS-c, a regulatory peptide encoded as a short ORF within the mitochondrial 12S rRNA gene. In cultured human cells, nutrient deprivation or reactive oxygen species exposure caused MOTS-c to translocate from the mitochondria to the nucleus, bind nuclear transcription factors, and promote expression of nuclear genes for resistance to metabolic stress (206).

Beyond mitochondrial proteins known to play dual roles in the mitochondria and nucleus, studies point to the importance of the mitochondria in modifying chromatin and nuclear DNA. Mitochondrial metabolism provides many of the requisite substrates for these epigenetic modifications, including acetyl-CoA, formate, ATP, and α-ketoglutarate. The perturbation of mitochondrial function results in altered epigenetic and transcriptional profiles within the nucleus (207, 208, 209, 210). For example, in glioblastoma cells, depletion of the outer mitochondrial membrane protein VDAC1, a key mediator of metabolic coordination between mitochondria and the rest of the cell, resulted in altered transcriptional patterns of nuclear genes involved in metabolic reprogramming and histone acetylation and methylation (207). In cultured human myoblasts, mitochondrial stress induced prosurvival changes to nuclear chromatin methylation (209). In other human cell lines, mtDNA depletion altered nuclear methylation and transcription to promote metabolic adaptations (211). In addition to these retrograde signaling mechanisms, metabolite sensing by mitochondrial proteins is an essential transcriptional regulation mechanism. Both yeast and human mtRNAP can sense a cell's metabolic state, specifically [NAD^+^]/[ATP] and [NADH]/[ATP] ratios, and regulate expression of NAD^+^/H capped mitochondrial RNAs in response (212). Further, the proximity of metabolic enzymes in pathways such as fatty acid metabolism and one-carbon metabolism to mtDNA raises the intriguing possibility of signaling between cellular metabolic status and mtDNA replication and transcription.

## Conclusions

The mitochondria are hubs for many critical processes, from ATP production to cell signaling and apoptosis. Their many cellular roles, multifaceted communication with the nucleus, and the vast array of pathologies associated with their dysfunction underscore the importance of a better understanding of mitochondrial functions and regulation. Although mtRNAP is architecturally simpler than the nuclear RNAPs, mtDNA transcription must respond to the cell's energy demands, continually changing and requiring coordination between nuclear and mitochondrial DNA transcription. Our understanding of mitochondrial transcription regulation in mammalian systems is growing, with recent research illuminating aspects of regulation by transcription factors, metabolite sensing, and nucleoid dynamics. Although this research area is of great interest, we have very little understanding of its biochemical and molecular basis, which is essential for enabling new therapeutics for the myriad of mitochondrial-related diseases.

Detailed studies of mitochondrial transcription and the interplay between the mitochondria and nucleus enable a deeper understanding of diseases and disorders caused by disrupted mitochondrial functions. There are increasing numbers of inherited mutations in mtDNA and nuclear genes for mitochondrial proteins associated with mitochondrial disorders involving defective ATP production, leading to diverse and often devastating effects on the heart, nervous system, and skeletal muscle (213). Examples of such inherited conditions include Leigh syndrome, Leber hereditary optic neuropathy, and Kearns–Sayre syndrome (214). Mitochondrial dysfunctions contribute to diabetes, obesity, and metabolic syndromes (215, 216). Mitochondria play diverse roles in cancer, such as providing energy and biosynthetic products for rapid proliferation, supporting metabolic adaptation to the tumor microenvironment, and regulating oncogenic signaling and apoptosis (217, 218). Mitochondria support cellular immune functions. However, dysfunctions in mitochondria or trauma could release immunogenic mtDNA and mtRNA in the cytosol or circulation to cause severe or chronic inflammation (219, 220). Mitochondrial genetics, metabolism, and inflammation significantly impact age-associated pathologies (221) and neurodegenerative diseases such as Alzheimer's and Parkinson's diseases (222, 223). We are hopeful that an increased biochemical understanding of mitochondrial functions will lead to better diagnostics and treatments for these conditions.

Here, we highlighted recent advances in understanding the structure, function, and regulation of mtDNA transcription. Parallel studies of yeast and human mtDNA transcription machinery have significantly been informative in revealing the basic transcription initiation mechanisms, adding clarity regarding the transcription factors' roles that critically control mtDNA transcription events. Despite this recent progress, many questions remain. We have limited information on the mechanism of various transcription steps, starting from assembly on specific promoters and culminating in the formation of a fully processed RNA transcript. We lack structures of the intermediate complexes in the initiation pathway and have limited knowledge of the dynamics of initiation, elongation, and termination reactions. A mechanistic framework is foundational for understanding how the mtDNA transcriptional machinery is regulated and controlled to sensitively respond to the cell's energy needs.

## References

[1] Dyall S.D., Brown M.T., Johnson P.J. (2004). Ancient invasions: from endosymbionts to organelles. Science.

[2] Gray M.W., Burger G., Lang B.F. (1999). Mitochondrial evolution. Science.

[3] Lang B.F., Gray M.W., Burger G. (1999). Mitochondrial genome evolution and the origin of eukaryotes. Annu. Rev. Genet.

[4] Andersson S.G., Karlberg O., Canback B., Kurland C.G. (2003). On the origin of mitochondria: a genomics perspective. Philos. Trans. R. Soc. Lond. B. Biol. Sci.

[5] Anderson S., Bankier A.T., Barrell B.G., de Bruijn M.H., Coulson A.R., Drouin J., Eperon I.C., Nierlich D.P., Roe B.A., Sanger F., Schreier P.H., Smith A.J., Staden R., Young I.G. (1981). Sequence and organization of the human mitochondrial genome. Nature.

[6] Foury F., Roganti T., Lecrenier N., Purnelle B. (1998). The complete sequence of the mitochondrial genome of Saccharomyces cerevisiae. FEBS Lett.

[7] Bendich A.J. (2010). The end of the circle for yeast mitochondrial DNA. Mol. Cell.

[8] Gerhold J.M., Aun A., Sedman T., Jõers P., Sedman J. (2010). Strand invasion structures in the inverted repeat of *Candida albicans* mitochondrial DNA reveal a role for homologous recombination in replication. Mol. Cell.

[9] Cermakian N., Ikeda T.M., Miramontes P., Lang B.F., Gray M.W., Cedergren R. (1997). On the evolution of the single-subunit RNA polymerases. J. Mol. Evol.

[10] Shadel G.S. (1999). Yeast as a model for human mtDNA replication. Am. J. Hum. Genet.

[11] Barshad G., Marom S., Cohen T., Mishmar D. (2018). Mitochondrial DNA transcription and its regulation: an evolutionary perspective. Trends Genet.

[12] Gustafsson C.M., Falkenberg M., Larsson N.G. (2016). Maintenance and expression of mammalian mitochondrial DNA. Annu. Rev. Biochem.

[13] Hillen H.S., Temiakov D., Cramer P. (2018). Structural basis of mitochondrial transcription. Nat. Struct. Mol. Biol.

[14] Bouda E., Stapon A., Garcia-Diaz M. (2019). Mechanisms of mammalian mitochondrial transcription. Protein Sci.

[15] Lipinski K.A., Kaniak-Golik A., Golik P. (2010). Maintenance and expression of the S. cerevisiae mitochondrial genome—from genetics to evolution and systems biology. Biochim. Biophys. Acta.

[16] da Cunha F.M., Torelli N.Q., Kowaltowski A.J. (2015). Mitochondrial retrograde signaling: triggers, pathways, and outcomes. Oxid. Med. Cell Longev.

[17] Guaragnella N., Coyne L.P., Chen X.J., Giannattasio S. (2018). Mitochondria-cytosol-nucleus crosstalk: learning from *Saccharomyces cerevisiae*. FEMS Yeast Res.

[18] Borst P. (1972). Mitochondrial nucleic acids. Annu. Rev. Biochem.

[19] Nicholls T.J., Minczuk M. (2014). In D-loop: 40 years of mitochondrial 7S DNA. Exp. Gerontol.

[20] Uchida A., Murugesapillai D., Kastner M., Wang Y., Lodeiro M.F., Prabhakar S., Oliver G.V., Arnold J.J., Maher L.J., Williams M.C., Cameron C.E. (2017). Unexpected sequences and structures of mtDNA required for efficient transcription from the first heavy-strand promoter. Elife.

[21] Montoya J., Gaines G.L., Attardi G. (1983). The pattern of transcription of the human mitochondrial rRNA genes reveals two overlapping transcription units. Cell.

[22] Chang D.D., Clayton D.A. (1984). Precise identification of individual promoters for transcription of each strand of human mitochondrial DNA. Cell.

[23] Lodeiro M.F., Uchida A., Bestwick M., Moustafa I.M., Arnold J.J., Shadel G.S., Cameron C.E. (2012). Transcription from the second heavy-strand promoter of human mtDNA is repressed by transcription factor A *in vitro*. Proc. Natl. Acad. Sci. U. S. A.

[24] Zollo O., Tiranti V., Sondheimer N. (2012). Transcriptional requirements of the distal heavy-strand promoter of mtDNA. Proc. Natl. Acad. Sci. U. S. A.

[25] Blumberg A., Rice E.J., Kundaje A., Danko C.G., Mishmar D. (2017). Initiation of mtDNA transcription is followed by pausing, and diverges across human cell types and during evolution. Genome Res.

[26] Litonin D., Sologub M., Shi Y., Savkina M., Anikin M., Falkenberg M., Gustafsson C.M., Temiakov D. (2010). Human mitochondrial transcription revisited: only TFAM and TFB2M are required for transcription of the mitochondrial genes *in vitro*. J. Biol. Chem.

[27] Ramachandran A., Basu U., Sultana S., Nandakumar D., Patel S.S. (2017). Human mitochondrial transcription factors TFAM and TFB2M work synergistically in promoter melting during transcription initiation. Nucleic Acids Res.

[28] Shutt T.E., Lodeiro M.F., Cotney J., Cameron C.E., Shadel G.S. (2010). Core human mitochondrial transcription apparatus is a regulated two-component system *in vitro*. Proc. Natl. Acad. Sci. U. S. A.

[29] Zollo O., Sondheimer N. (2017). Topological requirements of the mitochondrial heavy-strand promoters. Transcription.

[30] Turk E.M., Das V., Seibert R.D., Andrulis E.D. (2013). The mitochondrial RNA landscape of *Saccharomyces cerevisiae*. PLoS ONE.

[31] Gerhold J.M., Sedman T., Visacka K., Slezakova J., Tomaska L., Nosek J., Sedman J. (2014). Replication intermediates of the linear mitochondrial DNA of *Candida parapsilosis* suggest a common recombination based mechanism for yeast mitochondria. J. Biol. Chem.

[32] Biswas T.K. (1999). Nucleotide sequences surrounding the nonanucleotide promoter motif influence the activity of yeast mitochondrial promoter. Biochemistry.

[33] Osinga K.A., De Haan M., Christianson T., Tabak H.F. (1982). A nonanucleotide sequence involved in promotion of ribosomal RNA synthesis and RNA priming of DNA replication in yeast mitochondria. Nucleic Acids Res.

[34] Cheetham G.M., Jeruzalmi D., Steitz T.A. (1999). Structural basis for initiation of transcription from an RNA polymerase-promoter complex. Nature.

[35] Cheetham G.M., Steitz T.A. (1999). Structure of a transcribing T7 RNA polymerase initiation complex. Science.

[36] De Wijngaert B., Sultana S., Dharia C., Vanbuel H., Shen J., Vasilchuk D., Martinez S.E., Kandiah E., Patel S.S., Das K. (2020). Cryo-EM structures reveal transcription initiation steps by yeast mitochondrial RNA polymerase. bioRxiv.

[37] Hillen H.S., Morozov Y.I., Sarfallah A., Temiakov D., Cramer P. (2017). Structural basis of mitochondrial transcription initiation. Cell.

[38] Ringel R., Sologub M., Morozov Y.I., Litonin D., Cramer P., Temiakov D. (2011). Structure of human mitochondrial RNA polymerase. Nature.

[39] Schwinghammer K., Cheung A.C., Morozov Y.I., Agaronyan K., Temiakov D., Cramer P. (2013). Structure of human mitochondrial RNA polymerase elongation complex. Nat. Struct. Mol. Biol.

[40] Sousa R., Chung Y.J., Rose J.P., Wang B.C. (1993). Crystal structure of bacteriophage T7 RNA polymerase at 3.3 Å resolution. Nature.

[41] Ramachandran A., Nandakumar D., Deshpande A.P., Lucas T.P., R R.B., Tang G.Q., Raney K., Yin Y.W., Patel S.S. (2016). The yeast mitochondrial RNA polymerase and transcription factor complex catalyzes efficient priming of DNA synthesis on single-stranded DNA. J. Biol. Chem.

[42] Wanrooij S., Fuste J.M., Farge G., Shi Y., Gustafsson C.M., Falkenberg M. (2008). Human mitochondrial RNA polymerase primes lagging-strand DNA synthesis in vitro. Proc. Natl. Acad. Sci. U. S. A.

[43] Morozov Y.I., Agaronyan K., Cheung A.C., Anikin M., Cramer P., Temiakov D. (2014). A novel intermediate in transcription initiation by human mitochondrial RNA polymerase. Nucleic Acids Res.

[44] Yakubovskaya E., Guja K.E., Eng E.T., Choi W.S., Mejia E., Beglov D., Lukin M., Kozakov D., Garcia-Diaz M. (2014). Organization of the human mitochondrial transcription initiation complex. Nucleic Acids Res.

[45] Wang Y., Shadel G.S. (1999). Stability of the mitochondrial genome requires an amino-terminal domain of yeast mitochondrial RNA polymerase. Proc. Natl. Acad. Sci. U. S. A.

[46] Paratkar S., Deshpande A.P., Tang G.Q., Patel S.S. (2011). The N-terminal domain of the yeast mitochondrial RNA polymerase regulates multiple steps of transcription. J. Biol. Chem.

[47] Falkenberg M., Gaspari M., Rantanen A., Trifunovic A., Larsson N.G., Gustafsson C.M. (2002). Mitochondrial transcription factors B1 and B2 activate transcription of human mtDNA. Nat. Genet.

[48] McCulloch V., Seidel-Rogol B.L., Shadel G.S. (2002). A human mitochondrial transcription factor is related to RNA adenine methyltransferases and binds *S*-adenosylmethionine. Mol. Cell Biol.

[49] Jang S.H., Jaehning J.A. (1991). The yeast mitochondrial RNA polymerase specificity factor, MTF1, is similar to bacterial σ factors. J. Biol. Chem.

[50] Matsunaga M., Jaehning J.A. (2004). Intrinsic promoter recognition by a “core” RNA polymerase. J. Biol. Chem.

[51] Schubot F.D., Chen C.J., Rose J.P., Dailey T.A., Dailey H.A., Wang B.C. (2001). Crystal structure of the transcription factor sc-mtTFB offers insights into mitochondrial transcription. Protein Sci.

[52] Basu U., Mishra N., Farooqui M., Shen J., Johnson L.C., Patel S.S. (2020). The C-terminal tails of the mitochondrial transcription factors Mtf1 and TFB2M are part of an autoinhibitory mechanism that regulates DNA binding. J. Biol. Chem.

[53] Cotney J., Shadel G.S. (2006). Evidence for an early gene duplication event in the evolution of the mitochondrial transcription factor B family and maintenance of rRNA methyltransferase activity in human mtTFB1 and mtTFB2. J. Mol. Evol.

[54] Kaufman B.A., Durisic N., Mativetsky J.M., Costantino S., Hancock M.A., Grutter P., Shoubridge E.A. (2007). The mitochondrial transcription factor TFAM coordinates the assembly of multiple DNA molecules into nucleoid-like structures. Mol. Biol. Cell.

[55] Larsson N.G., Wang J., Wilhelmsson H., Oldfors A., Rustin P., Lewandoski M., Barsh G.S., Clayton D.A. (1998). Mitochondrial transcription factor A is necessary for mtDNA maintenance and embryogenesis in mice. Nat. Genet.

[56] Miyakawa I., Fumoto S., Kuroiwa T., Sando N. (1995). Characterization of DNA-binding proteins involved in the assembly of mitochondrial nucleoids in the yeast *Saccharomyces cerevisiae*. Plant Cell Physiol.

[57] Fisher R.P., Lisowsky T., Parisi M.A., Clayton D.A. (1992). DNA wrapping and bending by a mitochondrial high mobility group-like transcriptional activator protein. J. Biol. Chem.

[58] Ngo H.B., Kaiser J.T., Chan D.C. (2011). The mitochondrial transcription and packaging factor Tfam imposes a U-turn on mitochondrial DNA. Nat. Struct. Mol. Biol.

[59] Rubio-Cosials A., Sidow J.F., Jiménez-Menéndez N., Fernández-Millán P., Montoya J., Jacobs H.T., Coll M., Bernadó P., Solá M. (2011). Human mitochondrial transcription factor A induces a U-turn structure in the light strand promoter. Nat. Struct. Mol. Biol.

[60] Chakraborty A., Lyonnais S., Battistini F., Hospital A., Medici G., Prohens R., Orozco M., Vilardell J., Solá M. (2017). DNA structure directs positioning of the mitochondrial genome packaging protein Abf2p. Nucleic Acids Res.

[61] Kukat C., Davies K.M., Wurm C.A., Spåhr H., Bonekamp N.A., Kühl I., Joos F., Polosa P.L., Park C.B., Posse V., Falkenberg M., Jakobs S., Kühlbrandt W., Larsson N.G. (2015). Cross-strand binding of TFAM to a single mtDNA molecule forms the mitochondrial nucleoid. Proc. Natl. Acad. Sci. U. S. A.

[62] Fisher R.P., Clayton D.A. (1985). A transcription factor required for promoter recognition by human mitochondrial RNA polymerase: accurate initiation at the heavy- and light-strand promoters dissected and reconstituted in vitro. J. Biol. Chem.

[63] Dairaghi D.J., Shadel G.S., Clayton D.A. (1995). Addition of a 29 residue carboxyl-terminal tail converts a simple HMG box-containing protein into a transcriptional activator. J. Mol. Biol.

[64] Malarkey C.S., Bestwick M., Kuhlwilm J.E., Shadel G.S., Churchill M.E. (2012). Transcriptional activation by mitochondrial transcription factor A involves preferential distortion of promoter DNA. Nucleic Acids Res.

[65] Wong T.S., Rajagopalan S., Freund S.M., Rutherford T.J., Andreeva A., Townsley F.M., Petrovich M., Fersht A.R. (2009). Biophysical characterizations of human mitochondrial transcription factor A and its binding to tumor suppressor p53. Nucleic Acids Res.

[66] Ngo H.B., Lovely G.A., Phillips R., Chan D.C. (2014). Distinct structural features of TFAM drive mitochondrial DNA packaging versus transcriptional activation. Nat. Commun.

[67] Morozov Y.I., Temiakov D. (2016). Human mitochondrial transcription initiation complexes have similar topology on the light and heavy strand promoters. J. Biol. Chem.

[68] Kang D., Kim S.H., Hamasaki N. (2007). Mitochondrial transcription factor A (TFAM): roles in maintenance of mtDNA and cellular functions. Mitochondrion.

[69] Sohn B.-K., Basu U., Lee S.-W., Cho H., Shen J., Deshpande A., Johnson L.C., Das K., Patel S.S., Kim H. (2020). The dynamic landscape of transcription initiation in yeast mitochondria. Nat. Commun.

[70] Fisher R.P., Clayton D.A. (1988). Purification and characterization of human mitochondrial transcription factor 1. Mol. Cell Biol.

[71] Tang G.Q., Deshpande A.P., Patel S.S. (2011). Transcription factor-dependent DNA bending governs promoter recognition by the mitochondrial RNA polymerase. J. Biol. Chem.

[72] Schinkel A.H., Groot Koerkamp M.J., Teunissen A.W., Tabak H.F. (1988). RNA polymerase induces DNA bending at yeast mitochondrial promoters. Nucleic Acids Res.

[73] Velazquez G., Sousa R., Brieba L.G. (2015). The thumb subdomain of yeast mitochondrial RNA polymerase is involved in processivity, transcript fidelity and mitochondrial transcription factor binding. RNA Biol.

[74] Kim H., Tang G.Q., Patel S.S., Ha T. (2012). Opening-closing dynamics of the mitochondrial transcription pre-initiation complex. Nucleic Acids Res.

[75] Posse V., Gustafsson C.M. (2017). Human mitochondrial transcription factor B2 is required for promoter melting during initiation of transcription. J. Biol. Chem.

[76] Tang G.Q., Anand V.S., Patel S.S. (2011). Fluorescence-based assay to measure the real-time kinetics of nucleotide incorporation during transcription elongation. J. Mol. Biol.

[77] Tang G.Q., Paratkar S., Patel S.S. (2009). Fluorescence mapping of the open complex of yeast mitochondrial RNA polymerase. J. Biol. Chem.

[78] Velazquez G., Guo Q., Wang L., Brieba L.G., Sousa R. (2012). Conservation of promoter melting mechanisms in divergent regions of the single-subunit RNA polymerases. Biochemistry.

[79] Hillen H.S., Parshin A.V., Agaronyan K., Morozov Y.I., Graber J.J., Chernev A., Schwinghammer K., Urlaub H., Anikin M., Cramer P., Temiakov D. (2017). Mechanism of transcription anti-termination in human mitochondria. Cell.

[80] Paratkar S., Patel S.S. (2010). Mitochondrial transcription factor Mtf1 traps the unwound non-template strand to facilitate open complex formation. J. Biol. Chem.

[81] Gaspari M., Falkenberg M., Larsson N.G., Gustafsson C.M. (2004). The mitochondrial RNA polymerase contributes critically to promoter specificity in mammalian cells. EMBO J.

[82] Biswas T.K., Getz G.S. (1986). Nucleotides flanking the promoter sequence influence the transcription of the yeast mitochondrial gene coding for ATPase subunit 9. Proc. Natl. Acad. Sci. U. S. A.

[83] Deshpande A.P., Patel S.S. (2012). Mechanism of transcription initiation by the yeast mitochondrial RNA polymerase. Biochim. Biophys. Acta.

[84] Basu U., Lee S.W., Deshpande A., Shen J., Sohn B.K., Cho H., Kim H., Patel S.S. (2020). The C-terminal tail of the yeast mitochondrial transcription factor Mtf1 coordinates template strand alignment, DNA scrunching and timely transition into elongation. Nucleic Acids Res.

[85] Amiott E.A., Jaehning J.A. (2006). Mitochondrial transcription is regulated via an ATP “sensing” mechanism that couples RNA abundance to respiration. Mol. Cell.

[86] Deshpande A.P., Patel S.S. (2014). Interactions of the yeast mitochondrial RNA polymerase with the +1 and +2 promoter bases dictate transcription initiation efficiency. Nucleic Acids Res.

[87] Drakulic S., Wang L., Cuéllar J., Guo Q., Velázquez G., Martín-Benito J., Sousa R., Valpuesta J.M. (2014). Yeast mitochondrial RNAP conformational changes are regulated by interactions with the mitochondrial transcription factor. Nucleic Acids Res.

[88] Savkina M., Temiakov D., McAllister W.T., Anikin M. (2010). Multiple functions of yeast mitochondrial transcription factor Mtf1p during initiation. J. Biol. Chem.

[89] Smerdon S.J., Jager J., Wang J., Kohlstaedt L.A., Chirino A.J., Friedman J.M., Rice P.A., Steitz T.A. (1994). Structure of the binding site for nonnucleoside inhibitors of the reverse transcriptase of human immunodeficiency virus type 1. Proc. Natl. Acad. Sci. U. S. A.

[90] Sosunov V., Sosunova E., Mustaev A., Bass I., Nikiforov V., Goldfarb A. (2003). Unified two-metal mechanism of RNA synthesis and degradation by RNA polymerase. EMBO J.

[91] Yin Y.W., Steitz T.A. (2002). Structural basis for the transition from initiation to elongation transcription in T7 RNA polymerase. Science.

[92] Kapanidis A.N., Margeat E., Ho S.O., Kortkhonjia E., Weiss S., Ebright R.H. (2006). Initial transcription by RNA polymerase proceeds through a DNA-scrunching mechanism. Science.

[93] Tang G.Q., Roy R., Ha T., Patel S.S. (2008). Transcription initiation in a single-subunit RNA polymerase proceeds through DNA scrunching and rotation of the N-terminal subdomains. Mol. Cell.

[94] Durniak K.J., Bailey S., Steitz T.A. (2008). The structure of a transcribing T7 RNA polymerase in transition from initiation to elongation. Science.

[95] Revyakin A., Liu C., Ebright R.H., Strick T.R. (2006). Abortive initiation and productive initiation by RNA polymerase involve DNA scrunching. Science.

[96] Sainsbury S., Niesser J., Cramer P. (2013). Structure and function of the initially transcribing RNA polymerase II-TFIIB complex. Nature.

[97] Samanta S., Martin C.T. (2013). Insights into the mechanism of initial transcription in *Escherichia coli* RNA polymerase. J. Biol. Chem.

[98] Zhang Y., Feng Y., Chatterjee S., Tuske S., Ho M.X., Arnold E., Ebright R.H. (2012). Structural basis of transcription initiation. Science.

[99] Kulbachinskiy A., Mustaev A. (2006). Region 3.2 of the σ subunit contributes to the binding of the 3′-initiating nucleotide in the RNA polymerase active center and facilitates promoter clearance during initiation. J. Biol. Chem.

[100] Pupov D., Kuzin I., Bass I., Kulbachinskiy A. (2014). Distinct functions of the RNA polymerase α subunit region 3.2 in RNA priming and promoter escape. Nucleic Acids Res.

[101] Belogurov G.A., Artsimovitch I. (2019). The mechanisms of substrate selection, catalysis, and translocation by the elongating RNA polymerase. J. Mol. Biol.

[102] Bandwar R.P., Tang G.Q., Patel S.S. (2006). Sequential release of promoter contacts during transcription initiation to elongation transition. J. Mol. Biol.

[103] Tang G.Q., Roy R., Bandwar R.P., Ha T., Patel S.S. (2009). Real-time observation of the transition from transcription initiation to elongation of the RNA polymerase. Proc. Natl. Acad. Sci. U. S. A.

[104] Tahirov T.H., Temiakov D., Anikin M., Patlan V., McAllister W.T., Vassylyev D.G., Yokoyama S. (2002). Structure of a T7 RNA polymerase elongation complex at 2.9 Å resolution. Nature.

[105] Mangus D.A., Jang S.H., Jaehning J.A. (1994). Release of the yeast mitochondrial RNA polymerase specificity factor from transcription complexes. J. Biol. Chem.

[106] Minczuk M., He J., Duch A.M., Ettema T.J., Chlebowski A., Dzionek K., Nijtmans L.G., Huynen M.A., Holt I.J. (2011). TEFM (c17orf42) is necessary for transcription of human mtDNA. Nucleic Acids Res.

[107] Posse V., Shahzad S., Falkenberg M., Hällberg B.M., Gustafsson C.M. (2015). TEFM is a potent stimulator of mitochondrial transcription elongation *in vitro*. Nucleic Acids Res.

[108] Sultana S., Solotchi M., Ramachandran A., Patel S.S. (2017). Transcriptional fidelities of human mitochondrial POLRMT, yeast mitochondrial Rpo41, and phage T7 single-subunit RNA polymerases. J. Biol. Chem.

[109] Agaronyan K., Morozov Y.I., Anikin M., Temiakov D. (2015). Mitochondrial biology: replication-transcription switch in human mitochondria. Science.

[110] Yu H., Xue C., Long M., Jia H., Xue G., Du S., Coello Y., Ishibashi T. (2018). TEFM enhances transcription elongation by modifying mtRNAP pausing dynamics. Biophys. J.

[111] Baldacci G., Bernardi G. (1982). Replication origins are associated with transcription initiation sequences in the mitochondrial genome of yeast. EMBO J.

[112] Lee D.Y., Clayton D.A. (1998). Initiation of mitochondrial DNA replication by transcription and R-loop processing. J. Biol. Chem.

[113] Posse V., Al-Behadili A., Uhler J.P., Clausen A.R., Reyes A., Zeviani M., Falkenberg M., Gustafsson C.M. (2019). RNase H1 directs origin-specific initiation of DNA replication in human mitochondria. PLoS Genet.

[114] Sanchez-Sandoval E., Diaz-Quezada C., Velazquez G., Arroyo-Navarro L.F., Almanza-Martinez N., Trasviña-Arenas C.H., Brieba L.G. (2015). Yeast mitochondrial RNA polymerase primes mitochondrial DNA polymerase at origins of replication and promoter sequences. Mitochondrion.

[115] Xu B., Clayton D.A. (1996). RNA-DNA hybrid formation at the human mitochondrial heavy-strand origin ceases at replication start sites: an implication for RNA-DNA hybrids serving as primers. EMBO J.

[116] Jiang S., Koolmeister C., Misic J., Siira S., Kühl I., Silva Ramos E., Miranda M., Jiang M., Posse V., Lytovchenko O., Atanassov I., Schober F.A., Wibom R., Hultenby K., Milenkovic D. (2019). TEFM regulates both transcription elongation and RNA processing in mitochondria. EMBO Rep.

[117] Markov D.A., Savkina M., Anikin M., Del Campo M., Ecker K., Lambowitz A.M., De Gnore J.P., McAllister W.T. (2009). Identification of proteins associated with the yeast mitochondrial RNA polymerase by tandem affinity purification. Yeast.

[118] Markov D.A., Wojtas I.D., Tessitore K., Henderson S., McAllister W.T. (2014). Yeast DEAD box protein Mss116p is a transcription elongation factor that modulates the activity of mitochondrial RNA polymerase. Mol. Cell Biol.

[119] Jemt E., Persson O., Shi Y., Mehmedovic M., Uhler J.P., Dávila López M., Freyer C., Gustafsson C.M., Samuelsson T., Falkenberg M. (2015). Regulation of DNA replication at the end of the mitochondrial D-loop involves the helicase TWINKLE and a conserved sequence element. Nucleic Acids Res.

[120] Kruse B., Narasimhan N., Attardi G. (1989). Termination of transcription in human mitochondria: identification and purification of a DNA binding protein factor that promotes termination. Cell.

[121] Asin-Cayuela J., Gustafsson C.M. (2007). Mitochondrial transcription and its regulation in mammalian cells. Trends Biochem. Sci.

[122] Asin-Cayuela J., Schwend T., Farge G., Gustafsson C.M. (2005). The human mitochondrial transcription termination factor (mTERF) is fully active *in vitro* in the non-phosphorylated form. J. Biol. Chem.

[123] Guja K.E., Garcia-Diaz M. (2012). Hitting the brakes: termination of mitochondrial transcription. Biochim. Biophys. Acta.

[124] Yakubovskaya E., Mejia E., Byrnes J., Hambardjieva E., Garcia-Diaz M. (2010). Helix unwinding and base flipping enable human MTERF1 to terminate mitochondrial transcription. Cell.

[125] Terzioglu M., Ruzzenente B., Harmel J., Mourier A., Jemt E., López M.D., Kukat C., Stewart J.B., Wibom R., Meharg C., Habermann B., Falkenberg M., Gustafsson C.M., Park C.B., Larsson N.G. (2013). MTERF1 binds mtDNA to prevent transcriptional interference at the light-strand promoter but is dispensable for rRNA gene transcription regulation. Cell Metab.

[126] Shi Y., Posse V., Zhu X., Hyvärinen A.K., Jacobs H.T., Falkenberg M., Gustafsson C.M. (2016). Mitochondrial transcription termination factor 1 directs polar replication fork pausing. Nucleic Acids Res.

[127] Mulcair M.D., Schaeffer P.M., Oakley A.J., Cross H.F., Neylon C., Hill T.M., Dixon N.E. (2006). A molecular mousetrap determines polarity of termination of DNA replication in *E. coli*. Cell.

[128] Pandey M., Elshenawy M.M., Jergic S., Takahashi M., Dixon N.E., Hamdan S.M., Patel S.S. (2015). Two mechanisms coordinate replication termination by the *Escherichia coli* Tus-Ter complex. Nucleic Acids Res.

[129] Byrnes J., Garcia-Diaz M. (2011). Mitochondrial transcription: how does it end?. Transcription.

[130] Hess J.F., Parisi M.A., Bennett J.L., Clayton D.A. (1991). Impairment of mitochondrial transcription termination by a point mutation associated with the MELAS subgroup of mitochondrial encephalomyopathies. Nature.

[131] Sasarman F., Antonicka H., Shoubridge E.A. (2008). The A3243G tRNALeu(UUR) MELAS mutation causes amino acid misincorporation and a combined respiratory chain assembly defect partially suppressed by overexpression of EFTu and EFG2. Hum. Mol. Genet.

[132] Morimoto R., Locker J., Synenki R.M., Rabinowitz M. (1979). Transcription, processing, and mapping of mitochondrial RNA from grande and petite yeast. J. Biol. Chem.

[133] Bogenhagen D.F., Rousseau D., Burke S. (2008). The layered structure of human mitochondrial DNA nucleoids. J. Biol. Chem.

[134] Han S., Udeshi N.D., Deerinck T.J., Svinkina T., Ellisman M.H., Carr S.A., Ting A.Y. (2017). Proximity biotinylation as a method for mapping proteins associated with mtDNA in living cells. Cell Chem. Biol.

[135] Surovtseva Y.V., Shutt T.E., Cotney J., Cimen H., Chen S.Y., Koc E.C., Shadel G.S. (2011). Mitochondrial ribosomal protein L12 selectively associates with human mitochondrial RNA polymerase to activate transcription. Proc. Natl. Acad. Sci. U. S. A.

[136] Nouws J., Goswami A.V., Bestwick M., McCann B.J., Surovtseva Y.V., Shadel G.S. (2016). Mitochondrial ribosomal protein L12 is required for POLRMT stability and exists as two forms generated by alternative proteolysis during import. J. Biol. Chem.

[137] Zhang Q., Liang Z., Gao Y., Teng M., Niu L. (2017). Differentially expressed mitochondrial genes in breast cancer cells: potential new targets for anti-cancer therapies. Gene.

[138] Serre V., Rozanska A., Beinat M., Chretien D., Boddaert N., Munnich A., Rötig A., Chrzanowska-Lightowlers Z.M. (2013). Mutations in mitochondrial ribosomal protein MRPL12 leads to growth retardation, neurological deterioration and mitochondrial translation deficiency. Biochim. Biophys. Acta.

[139] Liu L., Sanosaka M., Lei S., Bestwick M.L., Frey J.H., Surovtseva Y.V., Shadel G.S., Cooper M.P. (2011). LRP130 protein remodels mitochondria and stimulates fatty acid oxidation. J. Biol. Chem.

[140] Lei S., Sun R.Z., Wang D., Gong M.Z., Su X.P., Yi F., Peng Z.W. (2016). Increased hepatic fatty acids uptake and oxidation by LRPPRC-driven oxidative phosphorylation reduces blood lipid levels. Front. Physiol.

[141] Cui J., Wang L., Ren X., Zhang Y., Zhang H. (2019). LRPPRC: a multifunctional protein involved in energy metabolism and human disease. Front. Physiol.

[142] Ekstrand M.I., Falkenberg M., Rantanen A., Park C.B., Gaspari M., Hultenby K., Rustin P., Gustafsson C.M., Larsson N.G. (2004). Mitochondrial transcription factor A regulates mtDNA copy number in mammals. Hum. Mol. Genet.

[143] Bedrat A., Lacroix L., Mergny J.L. (2016). Re-evaluation of G-quadruplex propensity with G4Hunter. Nucleic Acids Res.

[144] Falabella M., Fernandez R.J., Johnson F.B., Kaufman B.A. (2019). Potential roles for G-quadruplexes in mitochondria. Curr. Med. Chem.

[145] Lyonnais S., Tarrés-Solé A., Rubio-Cosials A., Cuppari A., Brito R., Jaumot J., Gargallo R., Vilaseca M., Silva C., Granzhan A., Teulade-Fichou M.P., Eritja R., Sola M. (2017). Corrigendum: The human mitochondrial transcription factor A is a versatile G-quadruplex binding protein. Sci. Rep.

[146] Blumberg A., Danko C.G., Kundaje A., Mishmar D. (2018). A common pattern of DNase I footprinting throughout the human mtDNA unveils clues for a chromatin-like organization. Genome Res.

[147] Falabella M., Kolesar J.E., Wallace C., de Jesus D., Sun L., Taguchi Y.V., Wang C., Wang T., Xiang I.M., Alder J.K., Maheshan R., Horne W., Turek-Herman J., Pagano P.J., St Croix C.M. (2019). G-quadruplex dynamics contribute to regulation of mitochondrial gene expression. Sci. Rep.

[148] Dong D.W., Pereira F., Barrett S.P., Kolesar J.E., Cao K., Damas J., Yatsunyk L.A., Johnson F.B., Kaufman B.A. (2014). Association of G-quadruplex forming sequences with human mtDNA deletion breakpoints. BMC Genomics.

[149] Sharma N., Pasala M.S., Prakash A. (2019). Mitochondrial DNA: epigenetics and environment. Environ. Mol. Mutagen.

[150] Mposhi A., Van der Wijst M.G., Faber K.N., Rots M.G. (2017). Regulation of mitochondrial gene expression, the epigenetic enigma. Front. Biosci. (Landmark Ed.).

[151] Patil V., Cuenin C., Chung F., Aguilera J.R.R., Fernandez-Jimenez N., Romero-Garmendia I., Bilbao J.R., Cahais V., Rothwell J., Herceg Z. (2019). Human mitochondrial DNA is extensively methylated in a non-CpG context. Nucleic Acids Res.

[152] Hao Z., Wu T., Cui X., Zhu P., Tan C., Dou X., Hsu K.W., Lin Y.T., Peng P.H., Zhang L.S., Gao Y., Hu L., Sun H.L., Zhu A., Liu J. (2020). *N*^6^-Deoxyadenosine methylation in mammalian mitochondrial DNA. Mol. Cell.

[153] Matsuda S., Yasukawa T., Sakaguchi Y., Ichiyanagi K., Unoki M., Gotoh K., Fukuda K., Sasaki H., Suzuki T., Kang D. (2018). Accurate estimation of 5-methylcytosine in mammalian mitochondrial DNA. Sci. Rep.

[154] Bellizzi D., D'Aquila P., Scafone T., Giordano M., Riso V., Riccio A., Passarino G. (2013). The control region of mitochondrial DNA shows an unusual CpG and non-CpG methylation pattern. DNA Res.

[155] Bianchessi V., Vinci M.C., Nigro P., Rizzi V., Farina F., Capogrossi M.C., Pompilio G., Gualdi V., Lauri A. (2016). Methylation profiling by bisulfite sequencing analysis of the mtDNA non-coding region in replicative and senescent endothelial cells. Mitochondrion.

[156] van der Wijst M.G., van Tilburg A.Y., Ruiters M.H., Rots M.G. (2017). Experimental mitochondria-targeted DNA methylation identifies GpC methylation, not CpG methylation, as potential regulator of mitochondrial gene expression. Sci. Rep.

[157] Dou X., Boyd-Kirkup J.D., McDermott J., Zhang X., Li F., Rong B., Zhang R., Miao B., Chen P., Cheng H., Xue J., Bennett D., Wong J., Lan F., Han J.J. (2019). The strand-biased mitochondrial DNA methylome and its regulation by DNMT3A. Genome Res.

[158] Dostal V., Churchill M.E.A. (2019). Cytosine methylation of mitochondrial DNA at CpG sequences impacts transcription factor A DNA binding and transcription. Biochim. Biophys. Acta Gene Regul. Mech.

[159] Liu Y.F., Zhu J.J., Yu Tian X., Liu H., Zhang T., Zhang Y.P., Xie S.A., Zheng M., Kong W., Yao W.J., Pang W., Zhao C.R., Tang Y.J., Zhou J. (2020). Hypermethylation of mitochondrial DNA in vascular smooth muscle cells impairs cell contractility. Cell Death Dis.

[160] Dinardo M.M., Musicco C., Fracasso F., Milella F., Gadaleta M.N., Gadaleta G., Cantatore P. (2003). Acetylation and level of mitochondrial transcription factor A in several organs of young and old rats. Biochem. Biophys. Res. Commun.

[161] King G.A., Hashemi Shabestari M., Taris K.H., Pandey A.K., Venkatesh S., Thilagavathi J., Singh K., Krishna Koppisetti R., Temiakov D., Roos W.H., Suzuki C.K., Wuite G.J.L. (2018). Acetylation and phosphorylation of human TFAM regulate TFAM-DNA interactions via contrasting mechanisms. Nucleic Acids Res.

[162] Lu B., Lee J., Nie X., Li M., Morozov Y.I., Venkatesh S., Bogenhagen D.F., Temiakov D., Suzuki C.K. (2013). Phosphorylation of human TFAM in mitochondria impairs DNA binding and promotes degradation by the AAA+ Lon protease. Mol. Cell.

[163] Wang K.Z., Zhu J., Dagda R.K., Uechi G., Cherra S.J., Gusdon A.M., Balasubramani M., Chu C.T. (2014). ERK-mediated phosphorylation of TFAM downregulates mitochondrial transcription: implications for Parkinson's disease. Mitochondrion.

[164] Weinberg F., Hamanaka R., Wheaton W.W., Weinberg S., Joseph J., Lopez M., Kalyanaraman B., Mutlu G.M., Budinger G.R., Chandel N.S. (2010). Mitochondrial metabolism and ROS generation are essential for Kras-mediated tumorigenicity. Proc. Natl. Acad. Sci. U. S. A.

[165] Matsushima Y., Goto Y., Kaguni L.S. (2010). Mitochondrial Lon protease regulates mitochondrial DNA copy number and transcription by selective degradation of mitochondrial transcription factor A (TFAM). Proc. Natl. Acad. Sci. U. S. A.

[166] Bostwick A.M., Moya G.E., Senti M.L., Basu U., Shen J., Patel S.S., Dittenhafer-Reed K.E. (2020). Phosphorylation of mitochondrial transcription factor B2 controls mitochondrial DNA binding and transcription. Biochem. Biophys. Res. Commun.

[167] Dittenhafer-Reed K.E., Richards A.L., Fan J., Smallegan M.J., Fotuhi Siahpirani A., Kemmerer Z.A., Prolla T.A., Roy S., Coon J.J., Denu J.M. (2015). SIRT3 mediates multi-tissue coupling for metabolic fuel switching. Cell Metab.

[168] Grimsrud P.A., Carson J.J., Hebert A.S., Hubler S.L., Niemi N.M., Bailey D.J., Jochem A., Stapleton D.S., Keller M.P., Westphall M.S., Yandell B.S., Attie A.D., Coon J.J., Pagliarini D.J. (2012). A quantitative map of the liver mitochondrial phosphoproteome reveals posttranslational control of ketogenesis. Cell Metab.

[169] Hebert A.S., Dittenhafer-Reed K.E., Yu W., Bailey D.J., Selen E.S., Boersma M.D., Carson J.J., Tonelli M., Balloon A.J., Higbee A.J., Westphall M.S., Pagliarini D.J., Prolla T.A., Assadi-Porter F., Roy S. (2013). Calorie restriction and SIRT3 trigger global reprogramming of the mitochondrial protein acetylome. Mol Cell.

[170] Hornbeck P.V., Zhang B., Murray B., Kornhauser J.M., Latham V., Skrzypek E. (2015). PhosphoSitePlus, 2014: mutations, PTMs and recalibrations. Nucleic Acids Res.

[171] Chatterjee A., Seyfferth J., Lucci J., Gilsbach R., Preissl S., Bottinger L., Martensson C.U., Panhale A., Stehle T., Kretz O., Sahyoun A.H., Avilov S., Eimer S., Hein L., Pfanner N. (2016). MOF acetyl transferase regulates transcription and respiration in mitochondria. Cell.

[172] Oran A.R., Adams C.M., Zhang X.Y., Gennaro V.J., Pfeiffer H.K., Mellert H.S., Seidel H.E., Mascioli K., Kaplan J., Gaballa M.R., Shen C., Rigoutsos I., King M.P., Cotney J.L., Arnold J.J. (2016). Multi-focal control of mitochondrial gene expression by oncogenic MYC provides potential therapeutic targets in cancer. Oncotarget.

[173] Li F., Wang Y., Zeller K.I., Potter J.J., Wonsey D.R., O'Donnell K.A., Kim J.W., Yustein J.T., Lee L.A., Dang C.V. (2005). Myc stimulates nuclearly encoded mitochondrial genes and mitochondrial biogenesis. Mol. Cell Biol.

[174] Scarpulla R.C. (2002). Transcriptional activators and coactivators in the nuclear control of mitochondrial function in mammalian cells. Gene.

[175] Gleyzer N., Vercauteren K., Scarpulla R.C. (2005). Control of mitochondrial transcription specificity factors (TFB1M and TFB2M) by nuclear respiratory factors (NRF-1 and NRF-2) and PGC-1 family coactivators. Mol. Cell Biol.

[176] Bruni F., Polosa P.L., Gadaleta M.N., Cantatore P., Roberti M. (2010). Nuclear respiratory factor 2 induces the expression of many but not all human proteins acting in mitochondrial DNA transcription and replication. J. Biol. Chem.

[177] Głombik K., Stachowicz A., Ślusarczyk J., Trojan E., Budziszewska B., Suski M., Kubera M., Lasoń W., Wędzony K., Olszanecki R., Basta-Kaim A. (2015). Maternal stress predicts altered biogenesis and the profile of mitochondrial proteins in the frontal cortex and hippocampus of adult offspring rats. Psychoneuroendocrinology.

[178] Bennett J.P., Keeney P.M. (2020). Alzheimer's and Parkinson's brain tissues have reduced expression of genes for mtDNA OXPHOS proteins, mitobiogenesis regulator PGC-1α protein, and mtRNA stabilizing protein LRPPRC (LRP130). Mitochondrion.

[179] She H., Yang Q., Shepherd K., Smith Y., Miller G., Testa C., Mao Z. (2011). Direct regulation of complex I by mitochondrial MEF2D is disrupted in a mouse model of Parkinson disease and in human patients. J. Clin. Invest.

[180] Sepuri N.B.V., Tammineni P., Mohammed F., Paripati A. (2017). Nuclear transcription factors in the mitochondria: a new paradigm in fine-tuning mitochondrial metabolism. Handb. Exp. Pharmacol.

[181] Leigh-Brown S., Enriquez J.A., Odom D.T. (2010). Nuclear transcription factors in mammalian mitochondria. Genome Biol.

[182] Marinov G.K., Wang Y.E., Chan D., Wold B.J. (2014). Evidence for site-specific occupancy of the mitochondrial genome by nuclear transcription factors. PLoS ONE.

[183] Davey R.A., Grossmann M. (2016). Androgen receptor structure, function and biology: from bench to bedside. Clin. Biochem. Rev.

[184] Yaşar P., Ayaz G., User S.D., Güpür G., Muyan M. (2017). Molecular mechanism of estrogen-estrogen receptor signaling. Reprod. Med. Biol.

[185] Bajpai P., Koc E., Sonpavde G., Singh R., Singh K.K. (2019). Mitochondrial localization, import, and mitochondrial function of the androgen receptor. J. Biol. Chem.

[186] Pronsato L., Milanesi L., Vasconsuelo A. (2020). Testosterone induces up-regulation of mitochondrial gene expression in murine C2C12 skeletal muscle cells accompanied by an increase of nuclear respiratory factor-1 and its downstream effectors. Mol. Cell Endocrinol.

[187] Chen J.Q., Yager J.D., Russo J. (2005). Regulation of mitochondrial respiratory chain structure and function by estrogens/estrogen receptors and potential physiological/pathophysiological implications. Biochim. Biophys. Acta.

[188] Klinge C.M. (2020). Estrogenic control of mitochondrial function. Redox Biol.

[189] Oeckinghaus A., Ghosh S. (2009). The NF-κB family of transcription factors and its regulation. Cold Spring Harb. Perspect. Biol.

[190] Mitchell T.J., John S. (2005). Signal transducer and activator of transcription (STAT) signalling and T-cell lymphomas. Immunology.

[191] Ivanova I.G., Perkins N.D. (2019). Hypoxia induces rapid, STAT3 and ROS dependent, mitochondrial translocation of RelA(p65) and IκBα. Biosci. Rep.

[192] Johnson R.F., Witzel I.I., Perkins N.D. (2011). p53-dependent regulation of mitochondrial energy production by the RelA subunit of NF-κB. Cancer Res.

[193] Macias E., Rao D., Carbajal S., Kiguchi K., DiGiovanni J. (2014). Stat3 binds to mtDNA and regulates mitochondrial gene expression in keratinocytes. J. Invest. Dermatol.

[194] Xu Y.S., Liang J.J., Wang Y., Zhao X.J., Xu L., Xu Y.Y., Zou Q.C., Zhang J.M., Tu C.E., Cui Y.G., Sun W.H., Huang C., Yang J.H., Chin Y.E. (2016). STAT3 undergoes acetylation-dependent mitochondrial translocation to regulate pyruvate metabolism. Sci. Rep.

[195] Burglin T.R. (1991). The TEA domain: a novel, highly conserved DNA-binding motif. Cell.

[196] Kaneko K.J., DePamphilis M.L. (2013). TEAD4 establishes the energy homeostasis essential for blastocoel formation. Development.

[197] Kumar R.P., Ray S., Home P., Saha B., Bhattacharya B., Wilkins H.M., Chavan H., Ganguly A., Milano-Foster J., Paul A., Krishnamurthy P., Swerdlow R.H., Paul S. (2018). Regulation of energy metabolism during early mammalian development: TEAD4 controls mitochondrial transcription. Development.

[198] Riscal R., Schrepfer E., Arena G., Cissé M.Y., Bellvert F., Heuillet M., Rambow F., Bonneil E., Sabourdy F., Vincent C., Ait-Arsa I., Levade T., Thibaut P., Marine J.C., Portais J.C. (2016). Chromatin-bound MDM2 regulates serine metabolism and redox homeostasis independently of p53. Mol. Cell.

[199] Arena G., Cisse M.Y., Pyrdziak S., Chatre L., Riscal R., Fuentes M., Arnold J.J., Kastner M., Gayte L., Bertrand-Gaday C., Nay K., Angebault-Prouteau C., Murray K., Chabi B., Koechlin-Ramonatxo C. (2018). Mitochondrial MDM2 regulates respiratory complex I activity independently of p53. Mol. Cell.

[200] Lettieri-Barbato D., Ioannilli L., Aquilano K., Ciccarone F., Rosina M., Ciriolo M.R. (2019). FoxO1 localizes to mitochondria of adipose tissue and is affected by nutrient stress. Metabolism.

[201] Celestini V., Tezil T., Russo L., Fasano C., Sanese P., Forte G., Peserico A., Lepore Signorile M., Longo G., De Rasmo D., Signorile A., Gadaleta R.M., Scialpi N., Terao M., Garattini E. (2018). Uncoupling FoxO3A mitochondrial and nuclear functions in cancer cells undergoing metabolic stress and chemotherapy. Cell Death Dis.

[202] Wen Y.A., Xiong X., Scott T., Li A.T., Wang C., Weiss H.L., Tan L., Bradford E., Fan T.W.M., Chandel N.S., Barrett T.A., Gao T. (2019). The mitochondrial retrograde signaling regulates Wnt signaling to promote tumorigenesis in colon cancer. Cell Death Differ.

[203] Han B., Izumi H., Yasuniwa Y., Akiyama M., Yamaguchi T., Fujimoto N., Matsumoto T., Wu B., Tanimoto A., Sasaguri Y., Kohno K. (2011). Human mitochondrial transcription factor A functions in both nuclei and mitochondria and regulates cancer cell growth. Biochem. Biophys. Res. Commun.

[204] Lee E.J., Kang Y.C., Park W.H., Jeong J.H., Pak Y.K. (2014). Negative transcriptional regulation of mitochondrial transcription factor A (TFAM) by nuclear TFAM. Biochem. Biophys. Res. Commun.

[205] Temelie M., Savu D.I., Moisoi N. (2018). Intracellular and intercellular signalling mechanisms following DNA damage are modulated by PINK1. Oxid. Med. Cell Longev.

[206] Kim K.H., Son J.M., Benayoun B.A., Lee C. (2018). The mitochondrial-encoded peptide MOTS-c translocates to the nucleus to regulate nuclear gene expression in response to metabolic stress. Cell Metab.

[207] Amsalem Z., Arif T., Shteinfer-Kuzmine A., Chalifa-Caspi V., Shoshan-Barmatz V. (2020). The mitochondrial protein VDAC1 at the crossroads of cancer cell metabolism: the epigenetic link. Cancers (Basel).

[208] Kopinski P.K., Janssen K.A., Schaefer P.M., Trefely S., Perry C.E., Potluri P., Tintos-Hernandez J.A., Singh L.N., Karch K.R., Campbell S.L., Doan M.T., Jiang H., Nissim I., Nakamaru-Ogiso E., Wellen K.E. (2019). Regulation of nuclear epigenome by mitochondrial DNA heteroplasmy. Proc. Natl. Acad. Sci. U. S. A.

[209] Mayorga L., Salassa B.N., Marzese D.M., Loos M.A., Eiroa H.D., Lubieniecki F., García Samartino C., Romano P.S., Roqué M. (2019). Mitochondrial stress triggers a pro-survival response through epigenetic modifications of nuclear DNA. Cell Mol. Life Sci.

[210] Sun X., St John J.C. (2018). Modulation of mitochondrial DNA copy number in a model of glioblastoma induces changes to DNA methylation and gene expression of the nuclear genome in tumours. Epigenetics Chromatin.

[211] Lozoya O.A., Martinez-Reyes I., Wang T., Grenet D., Bushel P., Li J., Chandel N., Woychik R.P., Santos J.H. (2018). Mitochondrial nicotinamide adenine dinucleotide reduced (NADH) oxidation links the tricarboxylic acid (TCA) cycle with methionine metabolism and nuclear DNA methylation. PLoS Biol.

[212] Bird J.G., Basu U., Kuster D., Ramachandran A., Grudzien-Nogalska E., Towheed A., Wallace D.C., Kiledjian M., Temiakov D., Patel S.S., Ebright R.H., Nickels B.E. (2018). Highly efficient 5′ capping of mitochondrial RNA with NAD^+^ and NADH by yeast and human mitochondrial RNA polymerase. Elife.

[213] Picard M., Wallace D.C., Burelle Y. (2016). The rise of mitochondria in medicine. Mitochondrion.

[214] Khan N.A., Govindaraj P., Meena A.K., Thangaraj K. (2015). Mitochondrial disorders: challenges in diagnosis & treatment. Indian J. Med. Res.

[215] Montgomery M.K. (2019). Mitochondrial dysfunction and diabetes: is mitochondrial transfer a friend or foe?. Biology (Basel).

[216] Prasun P. (2020). Mitochondrial dysfunction in metabolic syndrome. Biochim. Biophys. Acta Mol. Basis Dis.

[217] Roth K.G., Mambetsariev I., Kulkarni P., Salgia R. (2020). The mitochondrion as an emerging therapeutic target in cancer. Trends Mol. Med.

[218] Zong W.X., Rabinowitz J.D., White E. (2016). Mitochondria and cancer. Mol. Cell.

[219] Faas M.M., de Vos P. (2020). Mitochondrial function in immune cells in health and disease. Biochim. Biophys. Acta Mol. Basis Dis.

[220] Zhong F., Liang S., Zhong Z. (2019). Emerging role of mitochondrial DNA as a major driver of inflammation and disease progression. Trends Immunol.

[221] Chocron E.S., Munkácsy E., Pickering A.M. (2019). Cause or casualty: the role of mitochondrial DNA in aging and age-associated disease. Biochim. Biophys. Acta Mol. Basis Dis.

[222] Stanga S., Caretto A., Boido M., Vercelli A. (2020). Mitochondrial dysfunctions: a red thread across neurodegenerative diseases. Int. J. Mol. Sci.

[223] Wang W., Zhao F., Ma X., Perry G., Zhu X. (2020). Mitochondria dysfunction in the pathogenesis of Alzheimer's disease: recent advances. Mol. Neurodegener.

